# Genome-wide bioinformatic analyses predict key host and viral factors in SARS-CoV-2 pathogenesis

**DOI:** 10.1038/s42003-021-02095-0

**Published:** 2021-05-17

**Authors:** Mariana G. Ferrarini, Avantika Lal, Rita Rebollo, Andreas J. Gruber, Andrea Guarracino, Itziar Martinez Gonzalez, Taylor Floyd, Daniel Siqueira de Oliveira, Justin Shanklin, Ethan Beausoleil, Taneli Pusa, Brett E. Pickett, Vanessa Aguiar-Pulido

**Affiliations:** 1 University of Lyon, INSA-Lyon, INRA BF2l, Villeurbanne, France; 2 NVIDIA Corporation, Santa Clara, CA USA; 3 Department of Biology, University of Konstanz, Konstanz, Germany; 4 Centre for Molecular Bioinformatics, Department of Biology, University Of Rome Tor Vergata, Rome, Italy; 5 Amsterdam UMC, Amsterdam, The Netherlands; 6 Center for Neurogenetics, Weill Cornell Medicine, Cornell University, New York, NY USA; 7 Laboratoire de Biométrie et Biologie Evolutive, Université de Lyon, Université Lyon 1, CNRS UMR 5558, Villeurbanne, France; 8 Brigham Young University, Provo, UT USA; 9 Luxembourg Centre for Systems Biomedicine, Belvaux, Luxembourg; 10 Department of Computer Science, University of Miami, Coral Gables, FL USA

**Keywords:** SARS-CoV-2, RNA, Computational biology and bioinformatics, Transcriptomics, Viral infection

## Abstract

The novel betacoronavirus severe acute respiratory syndrome coronavirus 2 (SARS-CoV-2) caused a worldwide pandemic (COVID-19) after emerging in Wuhan, China. Here we analyzed public host and viral RNA sequencing data to better understand how SARS-CoV-2 interacts with human respiratory cells. We identified genes, isoforms and transposable element families that are specifically altered in SARS-CoV-2-infected respiratory cells. Well-known immunoregulatory genes including *CSF2, IL32, IL-6* and *SERPINA3* were differentially expressed, while immunoregulatory transposable element families were upregulated. We predicted conserved interactions between the SARS-CoV-2 genome and human RNA-binding proteins such as the heterogeneous nuclear ribonucleoprotein A1 (hnRNPA1) and eukaryotic initiation factor 4 (eIF4b). We also identified a viral sequence variant with a statistically significant skew associated with age of infection, that may contribute to intracellular host–pathogen interactions. These findings can help identify host mechanisms that can be targeted by prophylactics and/or therapeutics to reduce the severity of COVID-19.

## Abstract (web summary)

Ferrarini & Lal et al. developed a novel bioinformatic pipeline to explore how SARS-CoV-2 interacts with human respiratory cells using public available host gene expression and viral genome sequence data. Several human genes and proteins were predicted to play a role in the viral life cycle and the host response to SARS-CoV-2 infection.

## Introduction

In December of 2019, a novel betacoronavirus that was named severe acute respiratory syndrome coronavirus 2 (SARS-CoV-2) emerged in Wuhan, China^1,2^. This virus is responsible for causing the coronavirus disease of 2019 (COVID-19) and by 24 January 2021, it had already infected more than 95 million people worldwide, accounting for at least 2 million deaths^3^. The SARS-CoV-2 genome is phylogenetically distinct from the SARS-CoV and Middle East Respiratory Syndrome (MERS-CoV) betacoronaviruses that caused human outbreaks in 2002 and 2012, respectively^4,5^. Based on its high sequence similarity to a coronavirus isolated from bats^6^, SARS-CoV-2 is hypothesized to have originated from bat coronaviruses, potentially using pangolins as an intermediate host before infecting humans^7^.

It remains a global priority to develop effective treatments for COVID-19, including treatments that inhibit viral replication inside human cells. At the same time, it is critical to control the hyper-inflammatory state that is frequently caused by this infection^8^. It is therefore important to define the biological processes that occur early in infection, including the mechanisms of viral replication, transcription, and translation inside host cells, which can be targeted by therapeutics^9^, as well as host immune responses that can be modulated^8^. Although many aspects of SARS-CoV-2 infection may be shared with other respiratory viruses, it is particularly interesting to identify its specific molecular interactions with host cells, to explain the unique clinical and epidemiological features of COVID-19^10,11^. Further, the observation of heterogeneous immune responses in COVID-19 patients^12^ emphasizes the importance of identifying molecular responses to SARS-CoV-2, which are consistent across patients, and can therefore be targeted to develop widely applicable treatments.

SARS-CoV-2 enters human cells by binding to the angiotensin-converting enzyme 2 (ACE2) receptor^13^. Once inside the infected cell, components of the virus interact with host cell machinery. Coronaviruses have been shown to co-opt a diverse range of host factors for their life cycle, forming both protein–protein interactions and RNA–protein interactions with host factors^14,15^. Furthermore, viruses generally trigger a drastic host response during infection. A subset of these specific changes in gene regulation are associated with viral replication and, therefore, can be seen as potential drug targets. In addition, transposable element (TE) overexpression has been observed upon viral infection^16^ and TEs have been actively implicated in gene regulatory networks related to immunity^17^.

Recent studies have sought to understand the molecular interactions between SARS-CoV-2 and infected cells^18,19^, and some have quantified gene expression changes in patient samples or cultured lung-derived cells infected by SARS-CoV-2^20–22^. However, it remains important to contrast the effects of SARS-CoV-2 with those of other respiratory viruses. Furthermore, host factors such as TEs and genetic isoforms remain understudied in the context of SARS-CoV-2 infection. Here we aim to identify host factors, pathways, and processes that are altered in response to SARS-CoV-2 infection in human cells, in particular those that are unaffected by other respiratory infections. Moreover, although many previous studies have examined immune cells, we focused specifically on human airway epithelial cells, as they are the primary entry points for respiratory viruses and therefore constitute the first producers of inflammatory signals that, in addition to their antiviral activity, promote the initiation of the innate and adaptive immune responses.

We identified a signature of altered gene expression that is consistent across published datasets of SARS-CoV-2-infected human lung cells. We present extensive results from functional analyses (signaling pathway enrichment, biological functions, transcript isoform usage, and TE overexpression) of the genes differentially expressed during SARS-CoV-2 infection^22^, highlighting a consistent isoform switch of the *IL-6* gene in SARS-CoV-2-infected cell lines. We also analyzed viral genome sequences to predict specific interactions between the SARS-CoV-2 RNA genome and human proteins that may be involved in viral replication, transcription, or translation, and identified at least one viral sequence variation that is significantly associated with patient age in humans. Knowledge of these molecular and genetic mechanisms is important to understand SARS-CoV-2 pathogenesis and to improve the future development of effective prophylactic and therapeutic treatments.

## Results

**Fig. 1:**
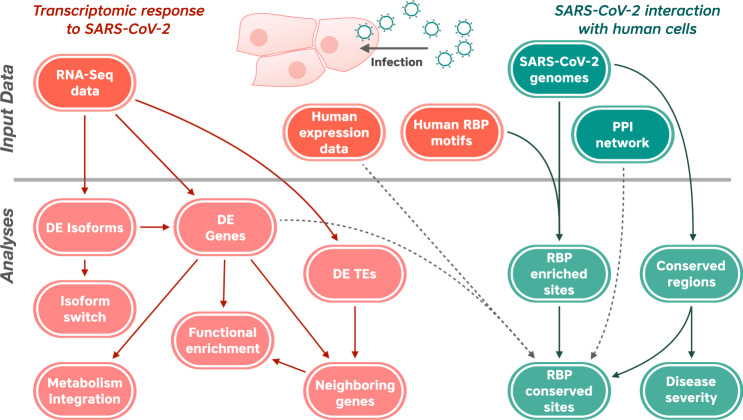
Overview of the bioinformatic workflow applied in this study. As indicated in orange, RNA-seq data from SARS-CoV-2-infected samples were used as the input to identify differentially expressed (DE) genes, isoforms, and transposable elements (TEs). DE genes were used to identify functional enrichment of deregulated genes and possible impacts on metabolism. Neighboring genes of differentially expressed TEs (DETEs) were analyzed to verify if TEs could serve as regulatory mechanisms of gene expression. In green, the complete genome of the SARS-CoV-2 virus was used to identify enrichment of RNA-binding protein (RBP) motifs. We also used all available sequenced genomes as of 11 November 2020, to detect conserved RBP motifs and possible links to disease severity.

We designed a comprehensive bioinformatics workflow to identify relevant host–pathogen interactions using a complementary set of computational analyses (Fig. 1). First, we carried out an exhaustive analysis of differential gene expression in human lung cells infected by SARS-CoV-2 or other respiratory viruses, identifying gene-, isoform-, and pathway-level responses, which specifically characterize SARS-CoV-2 infection. Second, we predicted putative interactions between the SARS-CoV-2 RNA genome and human RNA-binding proteins (RBPs). Third, we identified a subset of these human RBPs, which are also differentially expressed in response to SARS-CoV-2. Finally, we identified a viral sequence variant that could play a role in intracellular host–pathogen interaction.

### SARS-CoV-2 infection elicits a specific gene expression and pathway signature in human cells

**Fig. 2:**
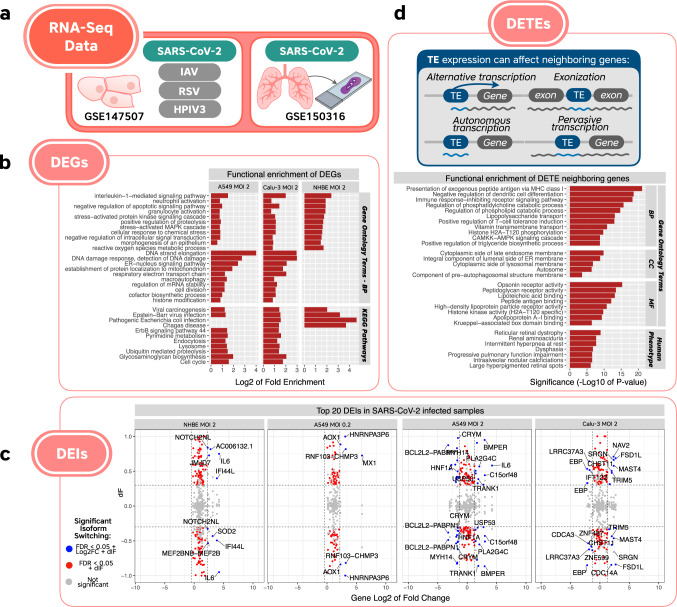
Overview of the RNA-seq-based results specific to SARS-CoV-2, which were not detected in the other viral infections (IAV, HPIV3, and RSV). **a** Representation of the RNA-seq studies used in our analyses. **b** A subset of non-redundant reduced terms consistently enriched in more than one SARS-CoV-2 cell line, which were not detected in the other viruses’ datasets. **c** Top 20 differentially expressed isoforms (DEIs) in SARS-CoV-2-infected samples. The *y*-axis denotes the differential usage of isoforms between conditions (i.e., difference in isoform fraction, dIF), whereas the *x*-axis represents the overall log2FC of the corresponding gene. Thus, DEIs also detected as differentially expressed genes (DEGs) by this analysis are depicted in blue. **d** Different manners by which transposable element (TE) family overexpression might be detected. Although TEs may be autonomously expressed, the old age of most TEs detected points toward either being part of a gene (exonization or alternative promoter) or a result of pervasive transcription. We report the functional enrichment for neighboring genes of differentially expressed TEs (DETEs) specifically upregulated in SARS-CoV-2 Calu-3 and A549 cells (MOI 2). Source data for Fig. 2 is provided in Supplementary Data 18.

We wanted to identify genes that were differentially expressed across multiple SARS-CoV-2-infected samples, while not significant in samples infected with other respiratory viruses. As a primary dataset, we selected GSE147507^22^ (Fig. 2a), which includes gene expression measurements from three cell lines derived from the human respiratory system (NHBE, A549, Calu-3) infected either with SARS-CoV-2, influenza A virus (IAV), respiratory syncytial virus (RSV), or human parainfluenza virus 3 (HPIV3). We also analyzed an additional dataset GSE150316 (Fig. 2a), which includes RNA sequencing (RNA-seq) extracted from formalin-fixed, paraffin-embedded (FFPE) histological sections of lung biopsies from COVID-19 deceased patients and healthy individuals. This second dataset encompasses a variable number (1–5) of postmortem lung biopsies per subject. The results coming from FFPE sections were less consistent, presumably due to the collection of biospecimens from different sites within the lung. Supplementary Data 1 provides details of all the samples included in our analyses.

We retrieved 41 differentially expressed genes (DEGs) that showed significant and consistent expression changes in at least three datasets from cell lines infected with SARS-CoV-2 and were not significantly affected in cell lines infected with other viruses within the same dataset. To these, we added 36 genes that showed significant and consistent expression changes in 2 of 4 cell line datasets infected with SARS-CoV-2 and at least 1 lung biopsy sample from a SARS-CoV-2 patient. The rationale behind these criteria was that results from FFPE sections were less reliable and, hence, were used only as supporting evidence where a gene was altered in at least two cell line samples. We further excluded four discordant genes that were upregulated in more than one cell line sample and downregulated in the biopsy samples. Thus, the final set consisted of 73 DEGs (Supplementary Data 2a): 53 upregulated and 20 downregulated, of which 41 had an absolute Log2FC > 1 in at least one dataset (selected genes from this list are shown in Table 1).

**Table 1:** Differentially expressed genes specific to SARS-CoV-2 infection.

Gene	Cell type and MOI				Also detected in biopsies
	A549	A549	Calu-3	NHBE	
	MOI 0.2	MOI 2			
*VNN2*		6.18	0.42	6.13	
*CSF2*		3.56	7.30	2.70	
*WNT7A*		4.99	0.79	0.45	
*PDZK1IP1*		1.72	0.70	2.28	
*SERPINA3*	0.49	1.39	0.77	1.44	Case 9
*RHCG*		1.51	2.02	1.33	
*IL32*		1.64	1.23	1.21	Case 1
*PDGFB*		1.91	1.75	1.00	
*ALDH1A3*		1.09	1.32	0.39	
*TLR2*		1.63	0.89	0.84	
*SERPINB1*		0.61	1.17	0.72	
*PRDM1*		0.82	3.49	0.59	
*MT-TN*		0.55	1.70	0.33	
*ATF4*		0.79	1.07	0.26	
*PTPN12*	0.48	0.97	1.23		
*DUSP16*	0.33	0.41	1.43		
*FKBP5*		−0.39		−0.36	Cases 1, 3, 8, 11
*DAP*		−0.18	−0.61		Case 1
*FECH*		−0.27	−0.36		Case 1
*MT-CYB*		−0.30	−0.26		Case 1, 8
*EIF4A1*		−0.33	−0.63		Case 1
*POLE4*	−0.23	−0.82	−1.24		
*DDX39A*	−0.23	−1.27	−0.54		
*CENPP*		−0.36	−0.40	−0.38	
*TMEM50B*		−0.48	−0.59	−0.53	
*HPS1*	−0.28	−0.31	−0.62		
*SNX8*	−0.30	−0.43	−0.56		

Log2 fold change for selected genes that showed significant up- or downregulation in SARS-CoV-2-infected samples (FDR-adjusted *p*-value < 0.05) and not in samples infected with the other viruses tested.

*MOI* multiplicity of infection.

*SERPINA3*, an antichymotrypsin that was proposed as a candidate inhibitor of viral replication^23^, was the only gene specifically upregulated in the four cell line datasets tested (Table 1). Other interesting upregulated genes were the amidohydrolase *VNN2*, the pro-fibrotic gene *PDGFB*, the β-interferon (IFN) regulator *PRDM1* and the proinflammatory cytokines *CSF2* and *IL32*. *FKBP5*, a known regulator of nuclear factor-κB (NF-κB) activity, was among the consistently downregulated genes. This set of genes represents a signature of host response specific to SARS-CoV-2 and may help to explain the specific clinical and epidemiological features of this disease. We also generated additional lists of DEGs that met different filtering criteria (Supplementary Data 2b, c and  3 for the complete DEG results for each dataset).

To better understand the underlying biological functions and molecular mechanisms associated with the observed DEGs, we performed a hypergeometric test to detect statistically significant overrepresented Gene Ontology (GO) terms^24^ among the DEGs having an absolute Log2FC > 1 in each dataset separately^24^.

Consistent with the findings of Blanco-Melo et al.^22^, GO enrichment analysis returned terms associated with immune system processes, response to cytokine, stress and viruses, and phosphatidylinositol 3-kinase (PI3K)/AKT signaling pathway, among others (see Supplementary Data 4 for complete results). In addition, we report 285 GO terms common to at least two cell line datasets infected with SARS-CoV-2 and absent in the response to other viruses (Fig. 2b and Supplementary Data 5a, b), including neutrophil and granulocyte activation, interleukin-1 (IL-1)-mediated signaling pathway, proteolysis, and stress-activated signaling cascades. We also detected 397 cell line-specific GO terms (76 in NHBE, 160 in A549, and 161 in Calu-3), which were not detected in the other viral datasets (Supplementary Data 5c). Our results show that each cell type regulates specific responses against SARS-CoV-2: A549-specific terms included vacuolar organization, endosome membrane, and protein export, whereas Calu-3-specific terms included oxidative phosphorylation, mitochondria, and cellular response to oxidative stress; NHBE cells had the majority of significant terms involved in cell chemotaxis and leukocyte-mediated immunity. One possible reason for these cell-specific responses is that each cell type expresses different levels of the viral receptor ACE2 (Supplementary Fig. 1).

Next, we wanted to pinpoint intracellular signaling pathways that may be modulated specifically during SARS-CoV-2 infection. A robust bootstrap-based signaling pathway impact analysis (SPIA) enabled us to identify 30 pathways, including many involved in the host immune response, which are significantly enriched among DEGs in at least one virus-infected cell line dataset (Supplementary Data 6). More importantly, we predicted four pathways to be specific to SARS-CoV-2 infection and observed that the significant pathways differ by cell type and multiplicity of infection (MOI). The significant results included only one term common to A549 (MOI 0.2) and Calu-3 cells (MOI 2), namely IFN-α/β signaling. In addition, we found the amoebiasis pathways (A549 cells, MOI 0.2) and the p75(NTR)-mediated and trka receptor signaling pathways (A549 cells, MOI 2) to be significantly impacted.

We also used a classic hypergeometric method as a complementary approach to our SPIA pathway enrichment analysis. Although there were generally higher numbers of significant results using this method, we observed that the vast majority of enriched terms (false discovery rate (FDR) < 0.05) described infections with various pathogens, innate immunity, metabolism, and cell cycle regulation (Supplementary Data 6). Interestingly, we were able to detect enriched Kyoto Encyclopedia of Genes and Genomes (KEGG) pathways common to at least two SARS-CoV-2-infected cell types and absent from the other virus-infected datasets (Fig. 2b). These included pathways related to infection, cell cycle, endocytosis, signaling pathways, cancer, and other diseases.

Our analyses highlight biological pathways in human lung cells that are altered specifically by SARS-CoV-2 infection, either in a cell-type-specific manner or consistent across cell types. This complements studies identifying pathway-level alterations in immune cells of COVID-19 patients^25–27^.

### SARS-CoV-2 infection results in altered lipid-related metabolic fluxes

To better understand how gene expression changes in response to virus infection impact human metabolism, we projected the transcriptomic data onto the human metabolic network^28^. This is an important analysis, as it can recover pathway-level changes that are not evident from analyzing dysregulated genes separately. This is based on the fact that the regulation of the entire metabolic pathways can be achieved by targeting few key enzymes via different regulatory mechanisms^29–31^. By integrating information of the metabolic network with differential expression, we can predict which connected pathways were most likely increased or decreased in viral infection^32^.

This analysis detected decreased fluxes in inositol phosphate metabolism in both A549 and Calu-3 cells infected with SARS-CoV-2 with an MOI of 2 (Supplementary Data 7). In addition, we detected an increased flux common to A549 and Calu-3 cell lines in reactive oxygen species detoxification, in accordance with previous terms recovered from functional enrichment analyses. Our analysis in A549 cells (MOI 2) also recovered decreased fluxes in several lipid pathways: fatty acid, cholesterol, sphingolipid, and glycerophospholipid, which have been shown as essential for the infection of multiple coronaviruses^33^. Overall, we were able to predict pathway-level changes that were not evident based only on DEGs, given that the control of key enzymes can be enough for the regulation of entire pathways.

### SARS-CoV-2 infection induced an isoform switch of genes associated with immunity and mRNA processing

We analyzed changes in transcript isoform expression and usage associated with SARS-CoV-2 infection, and predicted whether these changes might result in altered protein function.

We calculated isoform fraction (IF) values as the percentage of an individual isoform’s expression level relative to all other isoforms present within the parent gene’s expression level as presented in Eq. (1):

1 $${\mathrm{IF}}_{{\rm{isoform}}\; 1}=\frac{{{\rm{Isoform}}\; {{\rm{expression}}}}\; 1}{{\rm{Gene}}\ {\mathrm{expression}}\left({\rm{Isoform}}\; {{\rm{expression}}}\; 1+{\rm{isoform}}\; 2+\ldots +{\rm{isoform}}\; {n}\right)}$$

Differential isoform usage (difference in IF, dIF) is defined as the difference in the fraction of an isoform present between two conditions presented in Eq. (2):

2 $${\rm{dIF}}={\mathrm{IF}}_{{\rm{condition}}\; {2}}-{\mathrm{IF}}_{{\rm{condition}}\; {1}}$$

We identified isoforms experiencing a switch in usage ≥30% in absolute value (dIF ≥ |0.3|) across conditions and retrieved those with an FDR-adjusted *p*-value (*q*-value) < 0.05. Based on these criteria, we detected 3569 differentially expressed isoforms across all samples (Supplementary Table 1 and Supplementary Data 8). We performed biological consequence enrichment analysis to assess whether a particular consequence occurs more frequently than its opposite between conditions (Supplementary Fig. 2). For example, isoforms from A549 cells infected with RSV, IAV, and HPIV3 exhibited significant increases in nonsense-mediated decay (NMD) sensitivity and intron retention gain, while simultaneously exhibiting decreases in open reading frames (ORFs) and domains present. These conditions also displayed significant changes in splicing patterns, ranging from loss of exon skipping events, changes in usage of alternative transcription start and termination sites, and decreased alternative 5′- and 3′-splice sites (Supplementary Fig. 3).

In contrast, isoforms from SARS-CoV-2-infected samples displayed no significant global enrichment of biological consequences or alternative splicing events (Supplementary Figs. 2 and 3, respectively). However, nonsignificant trends (FDR-adjusted *p*-value > 0.05) indicated that certain transcripts in SARS-CoV-2 samples experienced decreases in ORF length, numbers of domains, coding capability, intron retention, and NMD (Supplementary Fig. 2). Although these trends were not significant on the genome-wide scale, they implicate that SARS-CoV-2 may trigger host machinery to target and aberrantly splice specific isoforms, leading to decreases in transcript length and, therefore, production of truncated proteins or alternative proteins.

To identify the specific isoforms affected by SARS-CoV-2 infection, we analyzed gene expression and isoform usage of individual isoforms in SARS-CoV-2 samples vs. controls. Results showed significant changes in gene expression and isoform usage at the individual gene level, with top-expressing isoforms associated with genes encoding cellular processes such as immune response and antiviral activity (*IFI44L*, *IL-6*, *MX1*, *TRIM5*), transcription and mRNA processing (*DDX10*, *HNRNPA3F6*, *JMJD7*, *ZNF487*, *ZNF599*), and cell cycle and survival (*BCL2L2-PABPN1*, *CDCA3*) (Fig. 2c, Supplementary Fig. 4, and Supplementary Data 8).

**Fig. 3:**
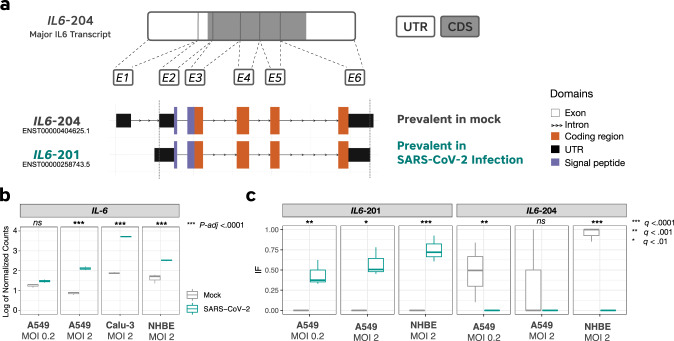
Isoform usage of *IL-6* transcripts in SARS-CoV-2-infected cells. **a** *IL6-204* is the major *IL-6* transcript and is composed of 6 exons, five (E2, E3, E4, E5, E6) containing coding sequences (CDS) and one (E1) containing exclusively a 5′-untranslated region (5′-UTR). Both isoforms (*IL6-204* and *IL6-201*) have the same protein-coding capability. The main difference between them is the absence of E1 in *IL6-201*, which is the major induced isoform upon SARS-CoV-2 infection. **b** Gene expression of *IL-6* in all SARS-CoV-2 cell line samples (A549 multiplicity of infection (MOI) 0.2 and 2; Calu-3 and NHBE MOI 2). Each boxplot represents three biological replicates and statistical testing was performed with DESeq2 (detailed in “Methods” section). Exact *p*-values are available in Supplementary Data 2. **c** Isoform usage switch between both isoforms in SARS-CoV-2-infected cell line samples. This figure shows that *IL6-204* is almost exclusively expressed in uninfected (mock) cells, whereas *IL6-201* is almost exclusively expressed in SARS-CoV-2-infected cells. Each boxplot represents three biological replicates and statistical testing was performed with IsoformSwitchAnalyzeR and exact *q*-values are available in Supplementary Data 8. Source data for Fig. 3 is provided in Supplementary Data 18.

We noticed that *IL-6*, a gene encoding a cytokine involved in acute and chronic inflammatory responses, displayed significant changes in both gene expression and isoform usage in SARS-CoV-2 infection. *IL-6* expression increased by two- to sixfold with an MOI of 2 (Fig. 3b). To date, the Ensembl Genome Reference Consortium has identified nine *IL-6* isoforms in humans, with the traditional transcript having six exons (*IL6-204*), five of which contain coding elements (Fig. 3a). NHBE cells expressed four known *IL-6* isoforms, whereas A549 cells expressed one unknown and six known isoforms (Supplementary Fig. 5). When evaluating the actual isoforms used across conditions, SARS-CoV-2-infected NHBE cells used three out of four isoforms observed, whereas SARS-CoV-2-infected A549 cells used all seven observed isoforms. For example, in the case of NHBE SARS-CoV-2 samples, the IF for the *IL6-201* isoform = 0.75, *IL6-204* = 0.05, *IL6-206* = 0.09, and *IL6-209* = 0.06, and the sum of these IF values = 0.95, or 95% usage of the *IL-6* gene relative to mock. SARS-CoV-2 samples (A549 MOI 0.2, A549 MOI 2, and NHBE MOI 2) exhibited exclusive usage of non-canonical isoform *IL6-201* (Fig. 3c) and, inversely, mock samples almost exclusively utilized the *IL6-204* transcript. In NHBE-infected cells, isoform *IL6-201* experienced a significant increase in usage (dIF = 0.75) and *IL6-204* a significant decrease in usage (dIF = −0.95) when compared to mock conditions. Similarly, isoform *IL6-201* in A549-infected cells experienced an increase in usage (dIF = 0.58), whereas uses of all other isoforms remained nonsignificant in comparison to mock conditions.

The *IL6-201* and *IL6-204* isoforms both contain five coding exons, and according to Ensembl, both are predicted to produce the same 212 amino acid protein product. The main difference between both isoforms is that *IL6-201* does not contain exon 1 (5′-untranslated region, 5′-UTR), which is present in *IL6-204*. The 5′-UTRs are traditionally involved in translational regulation, either promoting or inhibiting translation, depending upon the sequence and secondary RNA structure^34,35^ or modulating mRNA stability^36^. Thus, this isoform switch may be a mechanism to regulate IL-6 protein synthesis through control of translation rate and/or mRNA stability.

### Overexpression of TE families close to immune-associated genes upon SARS-CoV-2 infection

TEs are repeated DNA sequences that are able to spread across the genome, representing around two-thirds of the human genetic material^37^. TEs can be grouped into two different classes regarding their transposition mechanisms: (1) DNA elements, which are mobilized via a DNA molecule and make up around ~3% of the human genome^38^, and (2) retrotransposons, which have an RNA intermediate. Retrotransposons can be further divided into long-term repeat (LTR) elements, also named endogenous retroviruses, which account for ~8% of the human genome, or long and short interspersed nuclear elements (LINEs and SINEs) and SINE-VNTR-Alu elements, which lack LTRs and are the most abundant superfamilies in the human genome, accounting for around one-third of DNA sequences^38^. Although the human genome is bursting with TEs, most TE families are unable to transpose, either because they lost their transpositional machinery or because they have accumulated mutations that hinder their activity. There are only three TE families currently active in the human genome: LINE1, Alu (SINE) subfamily, and SVAs^39^. Nevertheless, the graveyard of dead TEs in the human genome has been repeatedly shown to regulate host gene expression, thus participating in key developmental and immune networks^40–42^. Therefore, searching for TE deregulation upon viral infection might shed light into activation of young TE families, but also pinpoint changes in gene regulatory networks.

To estimate the expression of TE families and their possible roles in SARS-CoV-2 infection, we mapped the RNA-seq reads against all annotated TE human families (see “Methods” section) and detected differentially expressed TE (DETE) families (Fig. 2d and Supplementary Data 9). We found 68 common TE families upregulated in SARS-CoV-2-infected A549 and Calu-3 cells (MOI 2), including 53 retrotransposons (24 LINEs, 27 LTRs, and two SINEs). It is important to note that none of the current transpositionally active human TE families were found to be upregulated in SARS-CoV-2-infected cells. From this list, we excluded all TE families detected in cells infected with the other viruses. This allowed us to identify 16 families that were specifically upregulated in Calu-3 and A549 cells infected with SARS-CoV-2, and not in the other viral infections. The 16 families identified are MER77B, MamRep4096, MLT2C2, PABL_A, Charlie9, MER34A, L1MEg1, LTR13A, L1MB5, MER11C, MER41B, LTR79, THE1D-int, MLT1I, MLT1F1, and MamRep137. Most of the TE families uncovered are ancient elements, and none are capable of transposing^43–45^. Eleven of the 16 TE families specifically upregulated in SARS-CoV-2-infected cells are LTR elements and include well-known TE immune regulators. For instance, MER41B (primate-specific TE family) is known to contribute to IFN-γ-inducible binding sites (bound by STAT1 and/or IRF1)^46,47^. Other LTR elements are also enriched in STAT1-binding sites (MER77B, LTR13, and MLT1l)^46^ or have been shown to act as cellular gene enhancers (LTR13A^48,49^).

In humans, TEs have been shown to accumulate in mammalian-specific gene regulatory sequences, such as within immunity-related gene transcripts^50^. Given that at least four TE families identified are well-known host–gene regulators, along with the general ability of TE families to impact nearby gene expression, we further investigated the functional enrichment of genes near these upregulated TE families (Supplementary Data 10). The GREAT method used for this analysis extends the regulatory domain of each annotated gene to 5 kb upstream and 1 kb downstream the transcription start site, as we still lack precise maps of gene regulatory regions^51^. We detected GO functional enrichment of several immunity-related terms (e.g., major histocompatibility complex (MHC) protein complex, antigen processing, regulation of dendritic cell differentiation, and T-cell tolerance induction), metabolism-related terms (such as regulation of phospholipid catabolic process), and, interestingly, a specific human phenotype term called “progressive pulmonary function impairment” (Fig. 2d).

Even though we did not limit our search only to neighboring genes which were also DE, we found several similar (and very specific) enriched terms in both analyses, for instance, related to endosomes, endoplasmic reticulum, and vitamin (cofactor) metabolism, among others. This result supports the idea that some responses during infection could be related to TE-mediated transcriptional regulation. Finally, when we searched for enriched terms related to each one of the 16 families separately, we also detected immunity-related enriched terms such as regulation of ILs, antigen processing, TGF-β receptor binding, and temperature homeostasis. It is important to note that given the old age of some of the TEs detected, overexpression might be associated with pervasive transcription or inclusion of TE copies within unspliced introns (Fig. 2d). In conclusion, we were able to demonstrate that 16 TE families are upregulated specifically upon SARS-CoV-2 infection, including four TE families previously shown to harbor STAT1/IRF1-binding sites, and are enriched close to immunity-related genes. Finally, to clearly pinpoint if such TE families are responsible for nearby gene regulation, future work should focus on TE-gene chimeric transcript searches (using long read RNA-seq or paired-end reads), mapping of regulatory sequences within TE copies using chromatin-related methods such as ATAC-seq, and deletion of TE copies followed by analysis of gene expression.

### The SARS-CoV-2 genome is enriched in binding motifs for 40 human proteins, most of them conserved across SARS-CoV-2 isolates

The SARS-CoV-2 virus possesses a positive-sense, single-stranded, monopartite RNA genome. Such viruses are well-known to co-opt host RBPs for diverse processes including viral replication, translation, viral RNA stability, assembly of viral protein complexes, and regulation of viral protein activity^14,15^. Therefore, we sought to predict host RBPs that may form functionally significant interactions with the SARS-CoV-2 genome.

**Fig. 4:**
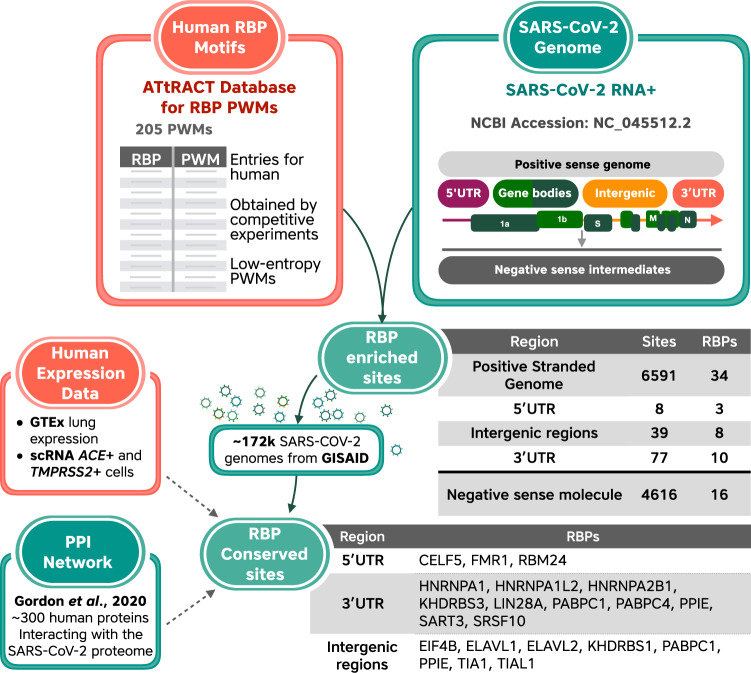
Workflow and selected results for the analysis of potential binding sites for human RNA-binding proteins (RBPs) in the SARS-CoV-2 genome. In orange, human RNA-binding protein (RBP) position weight matrices (PWMs) from the ATtRACT database were used as input to search for putative binding sites in the SARS-CoV-2 virus genome (green). Binding motifs of several RBPs were detected to be enriched/depleted within the positive-strand genome (containing genes, 5′- and 3′-untranslated regions (UTRs), and intergenic regions) and the negative-sense intermediates. Conserved RBP-binding sites were determined from the multiple sequence alignment of ~180k SARS-CoV-2 genomes available from GISAID. Finally, we included information from human gene expression data and protein–protein interaction networks for human and SARS-CoV-2 that are publicly available.

To do so, we first filtered the AtTRACT database^52^ to obtain a list of 102 human RBPs and 205 associated position weight matrices (PWMs) describing the experimentally determined sequence-binding preferences of these proteins. We then scanned the SARS-CoV-2 reference genome sequence to identify potential binding sites for these proteins. Figure 4 illustrates our analysis schema. We identified 99 human RBPs with a total of 11,897 potential binding sites in the SARS-CoV-2 positive-sense genome (Supplementary Data 11).

As the SARS-CoV-2 genome produces negative-sense intermediates as part of the replication process^53^, we also scanned the negative-sense genome sequence, where we found 11,333 potential binding sites for 96 RBPs (Supplementary Data 11).

To find RBPs whose binding sites occur in the SARS-CoV-2 genome more or less frequently than expected by chance, we repeatedly scrambled the genome sequence to create 1000 simulated genome sequences with an identical nucleotide composition to the SARS-CoV-2 genome sequence (30% A, 18% C, 20% G, 32% T). We used these 1000 simulated genomes to determine a background distribution of the number of binding sites found for a specific RBP. This allowed us to pinpoint RBPs with significantly more or fewer binding sites in the actual SARS-CoV-2 genome than expected based on the background distribution (two-tailed *z*-test, FDR-corrected *P* < 0.01). To retrieve RBPs whose motifs were enriched in specific genomic regions, we also repeated this analysis independently for the SARS-CoV-2 5′-UTR, 3′-UTR, intergenic regions, and for the negative-sense genome sequence. Motifs for 40 human RBPs were found to be enriched in at least one of the tested genomic regions, whereas motifs for 23 human RBPs were found to be depleted in at least one of the tested regions (Supplementary Data 12). Although experimental validation would be required to confirm the importance of these putative interactions, enrichment or depletion of binding sites for an RBP is suggestive that it may be beneficial or inhibitory, respectively, to viral replication.

We next examined whether any of the 6936 putative binding sites for these 40 enriched RBPs were conserved across SARS-CoV-2 isolates. We found that 6591 putative binding sites, representing 34 RBPs, were conserved across more than 95% of SARS-CoV-2 genome sequences in the GISAID database (≥171,953 out of 181,003 genomes). However, this is of limited significance, as the RBP-binding sites in coding regions are likely to be conserved due to evolutionary pressure on protein sequences rather than the RBP-binding ability. We therefore repeated this analysis focusing only on putative RBP-binding sites in the SARS-CoV-2 UTRs and intergenic regions. There were 124 putative RBP-binding sites for 21 enriched RBPs in the UTRs and intergenic regions. Of these, 50 putative RBP-binding sites for 17 RBPs were conserved in >95% of the available genome sequences; 6 in the 5′-UTR, 5 in the 3′-UTR, and 39 in intergenic regions (Supplementary Table 2).

Subsequently, we interrogated publicly available data to further investigate the putative SARS-CoV-2/RBP interactions (Supplementary Data 13). According to GTEx data^54^, 39 of the 40 enriched RBPs and all 23 of the depleted RBPs were expressed in human lung tissue. Furthermore, 31 of 40 enriched RBPs and 22 of 23 depleted RBPs were co-expressed with the *ACE2* and *TMPRSS2* receptor genes in single-cell RNA-seq data from human lung cells^55^, indicating that they are present in cells that are susceptible to SARS-CoV-2 infection. We next checked whether any of these RBPs have been reported to interact with SARS-CoV-2 proteins and found that human poly(A)-binding protein cytoplasmic 1 and 4 (PABPC1 and PABPC4, respectively) were reported to bind to the viral N protein in a recent study^18^. If this report is correct, these RBPs may interact with both the SARS-CoV-2 RNA and proteins. Finally, we combined these results with our analysis of differential gene expression to identify SARS-CoV-2-interacting RBPs that also show expression changes upon infection. The results of this analysis are summarized for selected RBPs in Table 2. Based on our computational analysis and existing literature, we highlight these enriched RBPs for their potential functional interaction with SARS-CoV-2 RNA.

**Table 2:** RNA-binding proteins (RBPs) predicted to interact with SARS-CoV-2.

RBP	DE analysis			Experimental evidence in human datasets				RBP-binding site prediction	
	A549 Log2FC	Calu-3 Log2FC	SARS-CoV-2-specific DEG	GTEx lung tissue (TPM)	scRNA	PPI map	Viral RNA binding	Conserved in SARS-CoV-2 genomes	Region
HNRNPA1		−0.32		331.336	TRUE		Yes	Yes	3′-UTR
HNRNPA2B1	−1.08	−0.29		539.829	TRUE		Yes	Yes	
PABPC1	0.72	0.44	Yes	448.025	TRUE	SARS-CoV-2 N protein	Yes		
PABPC4	0.30	−0.28		103.082	TRUE	SARS-CoV-2 N protein	Yes		
PPIE		−0.27		13.827	TRUE			Yes	
CELF5	0.56			0.079	TRUE			Yes	5′-UTR
FMR1		0.75		21.435	TRUE			Yes	
RBM24	0.34			1.412				Yes	
EIF4B	0.53	0.64		170.303	TRUE		Yes	Yes	Intergenic
ELAVL1		−0.31		27.440	TRUE		Yes	Yes	
PABPC1	0.72	0.44	Yes	448.025	TRUE	SARS-CoV-2 N protein	Yes	Yes	
PPIE		−0.27		13.827	TRUE			Yes	
TIA1	0.34	0.41	Yes	46.934	TRUE		Yes	Yes	
TIAL1	0.25			40.593				Yes	

Selected human RBPs whose putative binding sites are enriched in regions of the SARS-CoV-2 genome, along with experimental information. Log2 fold change is reported only for differentially expressed genes (DEGs) with FDR-adjusted *p*-value < 0.05. scRNA indicates whether the RBP is co-expressed with *ACE2* and *TMPRSS2* in single-cell RNA-seq data from human lung cells^55^; PPI Map indicates reported interaction with a SARS-CoV-2 viral protein^18^; viral RNA binding indicates RBPs experimentally found to interact with SARS-CoV-2 RNA in a human liver cell line^85,86^.

*UTR* untranslated region.

### Motif enrichment in SARS-CoV-2 differs from related coronaviruses

We repeated the above analysis to calculate the enrichment and depletion of RBP-binding motifs in the genomes of two related coronaviruses: (i) the SARS-CoV virus that caused the SARS outbreak in 2002–2003 and (ii) RaTG13, a bat coronavirus with a genome that is 96% identical with that of SARS-CoV-2^2,6^. We found that the pattern of enrichment and depletion of RBP-binding motifs in SARS-CoV-2 is different from that of the other two viruses (Supplementary Data 14 and 15). Specifically, the SARS-CoV-2 genome is uniquely enriched for binding sites of CELF5 in its 5′-UTR, PPIE in its 3′-UTR, and ELAVL1 in the viral negative-sense RNA molecule. Interestingly, ELAVL1 is a known stabilizer of RNA^56^, whereas CELF5 and PPIE participate in splicing^57,58^. Despite the high sequence identity between the two genomes, the single binding site for CELF5 on the SARS-CoV-2 5′-UTR is conserved in 95.8% of available SARS-CoV-2 genome sequences but absent in the 5′-UTR of RaTG13.

### A viral genome variant associated with host age

We used the meta-CATS software^59^ to test whether any viral sequence variants were associated with a change in disease severity, age, or biological sex in human hosts. We computed statistical correlations between 8079 complete SARS-CoV-2 genomes and the associated clinical metadata for each genome (e.g., severe, moderate, or mild disease; decade of age; and male or female). Briefly, this process calculates a *χ*^2^-score from a contingency table that contains the nucleotides present at each aligned position and the clinical metadata. The resulting *p*-value identifies positions that contain a statistically significant skew in the distribution of bases between the metadata categories. The alignment consisted of 30,649 nucleotide positions and 28,870 of these aligned positions contained at least one variant. We identified 3960 positions that contained at least one significant pairwise correlation with disease severity, 25 with patient age, and 883 with biological sex.

The FDR-corrected statistical results from this *χ*^2^-test of independence revealed a large number of nucleotide positions that showed statistically significant skew in the distribution of bases (Supplementary Data 16). However, further analysis revealed a low specificity for the vast majority of these results due primarily to the presence of ambiguous bases in a small number of the consensus genomes. This indicates that disease severity or infection of either biological sex of the patient cannot be solely attributed to a single viral variant.

In contrast, we identified a significant and specific skew in the distribution of bases between host age and aligned position 14,525 (cytosine to thymine at unaligned position 14,408 in the reference genome). The CCT to CTT codon variation (P323L in the RNA-dependent RNA polymerase (RdRP)) was found to significantly differ only when patients between 20 and 30 years old were compared against patients who were at least 85 years old (*p*-value < 0.04).

The distribution of bases between these two populations were 246C, 760T, and 3Y for the patients in their 20s, whereas the distribution for patients older than 85 years was 27C, 249T, and 7Y. These results show a 1 : 3 ratio of C to T in young patients and a ratio of ~1 : 9 of C to T in older patients. Two additional Pearson’s *χ*^2^-tests were subsequently performed, to account for the biological sex of the patients as covariates with age. These showed significant skew in the distribution of bases from viruses infecting 20- to 29-year-old males vs. >85-year-old males (*p*-values = 2.2 × 10^−16^), as well as in 20- to 29-year-old females vs. >85-year-old females (*p*-value = 3.2 × 10^−7^).

## Discussion

**Fig. 5:**
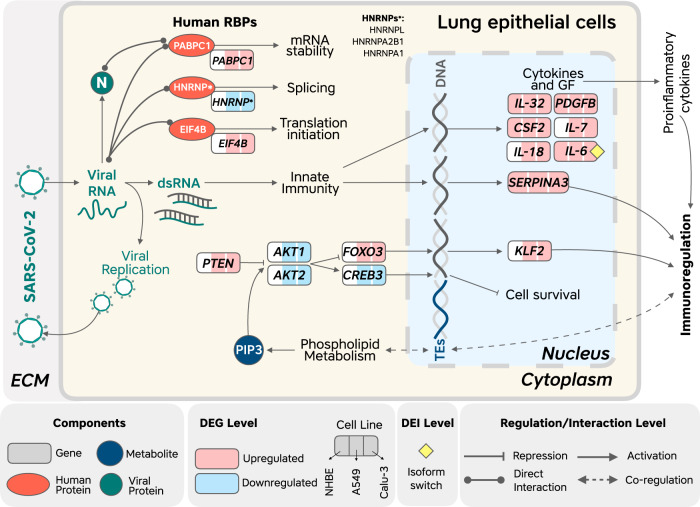
Overview of human factors specific to SARS-CoV-2 infection detected by our analyses. This figure includes human RNA-binding proteins (RBPs), whose binding sites are enriched and conserved in the SARS-CoV-2 genome but not in the genomes of related viruses, and gene isoforms and metabolites that are consistently altered in response to SARS-CoV-2 infection of lung epithelial cells but not in infection with the other tested viruses. ECM: extracellular matrix.

Here we report the results of a complementary panel of analyses that, together, enable a better understanding of host–pathogen interactions, which contribute to SARS-CoV-2 replication and pathogenesis in the human respiratory system. Figure 5 illustrates an overview of interesting host and viral factors detected in this work.

We performed a cross-dataset analysis of differential gene expression, highlighting specific genes that may play a role in the unique pathogenic features of COVID-19. Moreover, this analysis formed the basis for our subsequent dissection of pathway activity, metabolism, TEs, and regulatory activity in COVID-19. We discovered several known immunoregulators among the genes specifically and consistently altered in response to SARS-CoV-2, which points to distinct features of the immune response to this pathogen.

For example, *CSF2*, which encodes the granulocyte-macrophage colony stimulating factor (GM-CSF), was among the most highly upregulated genes in SARS-CoV-2-infected cells and is associated with tissue hyper-inflammation^60^. GM-CSF induces survival and activation in macrophages and neutrophils, and has been found at high levels in the blood of severe COVID-19 patients^61^, and several clinical trials are planned using agents that target GM-CSF or its receptor^62^. Another proinflammatory cytokine specifically upregulated in SARS-CoV-2-infected cells is *IL32*, which together with GM-CSF, promotes the release of tumor necrosis factor and IL-6 in a positive loop, and contributes to the cytokine storm^63^. In accordance, *IL-6* was upregulated in the three SARS-CoV-2-infected cell lines analyzed here. Moreover, not only upregulation but also a shift in isoform usage of *IL-6* was detected in three SARS-CoV-2-infected datasets. A shift in 5′-UTR usage in the presence of SARS-CoV-2 may be attributed to indirect host cell signaling cascades that trigger changes in transcription and splicing activity, which could also explain the overall increase in *IL-6* expression.

Aberrant isoform usage and splicing have previously been associated with the human antiviral response, cancer, and neurodegenerative diseases^64–66^; moreover, a recent study found that SARS-CoV-2 infection alters isoform usage of the *ERAP2* gene^67^. However, to our knowledge, ours is the first genome-wide analysis of the effect of SARS-CoV-2 on isoform usage. Additional experiments are needed to validate the effect of the *IL6-201* isoform on IL-6 protein activity in SARS-CoV-2-infected lung tissues. However, elevated IL-6 was observed in more than half of COVID-19 patients^68^ and was associated with COVID-19 complications, progression and poor prognosis, respiratory failure, sepsis, and mortality risk^69–73^. Our observations suggest the possibility that isoform switching, as well as upregulation of gene expression, may contribute to this IL-6 elevation. Although clinical trials evaluating IL-6 inhibitors in immune-based therapies exist^74^, the value in using IL-6 activity, and by extension all relevant isoforms, as a prognostic tool in determining severity and disease progression in SARS-CoV-2-infected patients is apparent.

SERPINA3, an essential enzyme in the regulation of leukocyte proteases, is induced by cytokines^75^ and has been proposed to inhibit viral replication^23^. This was the only gene consistently upregulated in all cell line samples infected with SARS-CoV-2 and absent from the other virus-infected datasets in this study. The *VNN2* gene was also upregulated in our analysis. Vanins are involved in proinflammatory and oxidative processes, and VNN2 plays a role in neutrophil migration by regulating b2 integrin^76^. Downregulated genes included *SNX8*, which has been reported in RNA virus-triggered induction of antiviral genes^23,77^, and *FKBP5*, a regulator of NF-κB activity^78^. These results suggest that SARS-CoV-2 tends to indirectly target specific genes involved in genome replication and host antiviral immune response without eliciting a global change in cellular transcript processing or protein production.

One of the first and most important innate antiviral responses is the production of type I IFN. This induces hundreds of IFN-stimulated genes, which limit virus spread and infection. Expression of SARS-CoV-2 proteins has previously been reported to inhibit the type I IFN signaling pathway^79^. Our signaling pathway analysis supported this by showing that type I IFN response was greatly impacted upon SARS-CoV-2 infection. We also observed elevated expression of *PRDM1* (Blimp-1) in SARS-CoV-2-infected cells, which may contribute to the critical regulation of IFN signaling cascades. Interestingly, the TE family LTR13, which was also upregulated, is enriched in *PRDM1* binding sites^80^. Therefore, it is possible that regulatory factors involved in IFN and immune response in SARS-CoV-2 infection could be partially attributed to TE transcriptional activation. Similarly, we detected upregulation of several immunoregulatory TE families in SARS-CoV-2-infected cells. The MER41B family, for instance, is known to contribute to IFN-γ-inducible binding sites (bound by STAT1 and/or IRF1). Functional enrichment of nearby genes was in accordance with these findings, as several immunity-related terms were enriched along with “progressive pulmonary impairment.”

In parallel, TEs seem to be co-regulated with phospholipid metabolism, which directly affects the PI3K/AKT signaling pathway, central to the immune response. Alterations in phospholipid metabolism and the PI3K/AKT pathway were detected in our metabolic flux analysis and functional enrichment analysis, respectively. A recent screen for host genes required for SARS-CoV-2 infection identified three members from the PI3K pathway^19^. In addition, phosphatidylinositol metabolic processes are important for the infection of multiple coronaviruses^33^ and it is well-known that lipid metabolism is essential throughout the life cycle of several viruses^81,82^. Moreover, both glycerophospholipids and fatty acids were reported to be significantly dysregulated in COVID-19 patients^83^. Finally, alteration of fatty acid metabolites in COVID-19 patients was highly correlated with IL-6 levels^84^, showing the potential of genome-wide complementary approaches to better understand this complex disease.

RBPs are likely candidates for host factors involved in the response of human cells to SARS-CoV-2, as well as viral manipulation of host machinery. During preparation of this study, two experimental studies^85,86^ reported hundreds of proteins that interact with SARS-CoV-2 RNA in human liver-derived cell lines. Encouragingly, they validated binding of several candidate proteins highlighted by our analysis (Table 2 and Supplementary Data 13). However, they did not identify the specific sites where these proteins bind to viral RNA and a deeper understanding of which RBPs promote or inhibit viral activity remains necessary. Our analysis complements these studies by (1) identifying putative binding sites for each protein on the viral genome, (2) identifying proteins whose binding sites were significantly enriched or depleted in the viral genome, and (3) identifying potential binding sites that are conserved and specific to SARS-CoV-2. We suggest that these proteins are likely to include functionally important interactions and should be the focus of experimental studies.

One of the RBPs whose potential binding sites are enriched and conserved in the SARS-CoV-2 genome is eIF4b, suggesting that SARS-CoV-2 viral protein translation could be eIF4b dependent. We also detected upregulation of the *EIF4B* gene in A549 and Calu-3 cells, which might indicate that this protein is sequestered by the virus and, therefore, cells need to increase its production. Another conserved RBP, which was also upregulated in infected cells, is the PABPC1, which is involved in mRNA stability and translation. PABPC1 has been implicated in multiple viral infections; it is modulated to inhibit host protein translation, promoting viral RNA access to the host translational machinery^87^. Interestingly, PABPC1 and PABPC4 have been reported to interact with the SARS-CoV-2 N protein, which stabilizes the viral genome^18^. This raises the possibility that the viral genome, N protein, and human PABP proteins may participate in a joint protein–RNA complex that assists in viral genome stability, replication, and/or translation^87–91^.

Binding motifs for hnRNPA1 were enriched specifically in the 3′-UTR of SARS-CoV-2, even though they were depleted in the genome overall. hnRNPA1 interacts with 3′-UTRs of other coronaviruses and participates in transcription and replication of the murine hepatitis virus^92–94^. The *hnRNPA1* gene, along with *hnRNPA2B1*, was downregulated in Calu-3 cells and, in contrast to the previous examples of upregulated genes, could denote a response from human cells to control viral replication.

Finally, we identified a significant association between a viral sequence variant and age of the host. The P323L mutation in the RdRP was previously shown to be associated with changes in geographical location of the viral strain^95^, although not with the age of the patient. It is possible that intracellular characteristics associated with senescence favor one allele in the polymerase over the other, such as stabilizing the interaction between the RdRp and the viral nsp8 proteins^96,97^. Such possibilities are consistent with previous indications that host cellular factors are critical to SARS-CoV-2 sequence evolution^98^. Our statistical analysis may contain sources of bias that are not limited to the number of genome sequences being collected earlier vs. later in the pandemic and the availability of genomes lacking complete clinical metadata. Although we examined patient sex as a possible covariate with age, it is impossible to account for all possible covariates due to the lack of data at the current time. Additional annotated datasets, as well as lab experiments are required to better elucidate the effect(s) of such viral sequence variants on the host response.

In conclusion, we envision that applying this workflow will yield important mechanistic insights in future analyses on emerging pathogens and we provide all source code freely for future use. Similarly, we expect that these findings will give rise to future studies that elucidate the underlying mechanism(s) responsible for such host–pathogen interactions. Modulating the host components of these mechanisms can aid in the selection of host-based drug targets, prophylactics, and/or therapeutics to reduce virus infection and replication with minimal adverse effects in humans.

## Methods

### RNA-seq data processing and differential expression analysis

Two datasets were downloaded from the NCBI Gene Expression Omnibus (GEO) database. The first dataset, GSE147507^22^, includes gene expression measurements from three cell lines derived from the human respiratory system (NHBE, A549, Calu-3) infected either with SARS-CoV-2, IAV, RSV, or HPIV3. The second dataset, GSE150316, includes RNA-seq extracted from FFPE histological sections of lung biopsies from COVID-19 deceased patients and healthy individuals. Supplementary Data 1 describes these datasets in detail.

For the first dataset (GSE147507), data were downloaded from Sequence Read Archive (SRA) using sra-tools (v2.10.8; https://github.com/ncbi/sra-tools) and transformed to FASTQ with fastq-dump. FastQC (v0.11.9; https://github.com/s-andrews/FastQC) and MultiQC (v1.9)^99^ were employed to assess the quality of the data and the need to trim reads and/or remove adapters. Selected datasets were mapped to the human reference genome (GENCODE Release 19, GRCh37.p13) using STAR (v.2.7.3a)^100^. Alignment statistics were used to determine which datasets should be included in subsequent steps. The resulting SAM files were converted to BAM files employing samtools (v1.9)^101^. Next, read quantification was performed using StringTie (v2.1.1)^102^ and the output data were post-processed with an auxiliary Python script provided by the same developers to produce files ready for subsequent downstream analyses. Expression was quantified for 57,820 genes based on GENCODE Release 19. For the second gene expression dataset (GSE150316), raw counts for 59,091 genes were directly downloaded from GEO.

DESeq2 (v1.26.0)^103^ was used in both cases to identify DEGs. Finally, an exploratory data analysis was carried out based on the transformed values obtained after applying the variance stabilizing transformation^104^ implemented in the vst() function of DESeq2^103^. Principal component analysis (PCA) was performed on the samples to evaluate the main sources of variation in the data and to remove outlier samples. Based on the PCA plots, no obvious outliers were detected in GSE147507; however, four entire samples (Cases 4, 6, 7, and 10) along with replicate 2 from Case 5 were detected as outliers and discarded from GSE150316 (Supplementary Fig. 6 and Supplementary Data 1).

### GO enrichment analysis

The DEGs produced by DESeq2 with an absolute Log2FC > 1 and FDR-adjusted *p*-value < 0.05 were used as input to a general GO enrichment analysis^105^. Each term was subjected to a hypergeometric test from the GOstats package (v2.54.0)^106^ and the *p*-values were corrected for multiple hypothesis testing, employing the Bonferroni method^107^. GO terms with a significant adjusted *p*-value < 0.05 were reduced to representative non-redundant terms with the use of REVIGO^108^.

### Host signaling pathway enrichment

The DEG lists produced by DESeq2 with an absolute Log2FC > 1 and FDR-adjusted *p*-value < 0.05 were used as input to the SPIA algorithm to identify significantly affected pathways from the R graphite library^109,110^. Pathways with Bonferroni-adjusted *p*-values < 0.05 were included in downstream analyses. The significant results for all comparisons from publicly available data from KEGG, Reactome, Panther, BioCarta, and NCI were then compiled to facilitate downstream comparison. Hypergeometric pathway enrichments were performed employing the Database for Annotation, Visualization, and Integrated Discovery (DAVID, v6.8)^111^.

### Integration of transcriptomic analysis with human metabolic network

To predict increased or decreased fluxes of reactions, we projected the transcriptomic data onto the human reconstructed metabolic network Recon (v2.2)^28^. This can be done based on the fact that the metabolic network includes gene-protein-reaction associations (GPRs), which in turn are easily mapped to the transcriptomic differential expression data. This, however, should not be seen as a quantitative measurement of fluxes, but rather as an indication of an activation or inactivation of certain parts of the network. First, we ran EBSeq (v3.12)^112^ on the gene count matrix generated in the previous steps. EBSeq returns the Posterior Probabilities of each gene being Differentially Expressed (PPDE) as well as the log2 fold changes. Then, we used the output of EBSeq as input to the Moomin method^32^ using default parameters. The Moomin method recovers topologically connected pathways predicted to be activated or inactivated based on the expression changes of corresponding GPRs included in the metabolic network. As there is usually not only one solution for a given differential expression dataset, a high number of solutions should be enumerated to construct a consensus solution. We enumerated 500 topological solutions for each of the datasets tested.

### Isoform analysis

Using transcript quantification data from StringTie as input, we identified isoform switching events and their predicted functional consequences with the IsoformSwitchAnalyzeR R package (v1.11.3)^113^.

To calculate differential activity between samples, isoform usage is measured by the IF value, which quantifies the individual isoform expression level relative to the parent gene’s expression level as previously presented in Eq. (1):

$${\mathrm{IF}}_{{\rm{isoform}}\; 1}=\frac{{\rm{{Isoform}}\; {\rm{expression}}}\; 1}{{\rm{Gene}}\ {\mathrm{expression}}\left({\rm{isoform}}\; {\rm{expression}}\; 1+{\rm{isoform}}\; 2+\ldots {\rm{isoform}}\; {n}\right)}$$

By proxy, the dIF between samples measures the effect size between conditions and is calculated as previously presented in Eq. (2):

$${\rm{dIF}}={\mathrm{IF}}_{{\rm{condition}}\; {2}}-{\mathrm{IF}}_{{\rm{condition}}\; 1}$$

dIF was measured on a scale of 0 to 1, with 0 = no (0%) change in usage between conditions and 1 = complete (100%) change in usage. The sum of dIF values for all isoforms associated with one gene is equal to 1. We next filtered for isoforms that experienced >30% switch in usage (dIF ≥ |0.3|) and had an FDR-corrected *p*-value cutoff of <0.05 (*q*-value < 0.05), which we define as “significant isoforms” for the remainder of the Methods.

Following filtering for these significant isoforms, we predicted their coding capabilities, protein structure stability, peptide signaling, and shifts in protein domain usage using The Coding-Potential Assessment Tool^114^, IUPred2^115^, SignalP^116^, and Pfam tools^117^, respectively. These external analyses were imported back into IsoformSwitchAnalyzeR and were used for downstream biological consequence and alternative splicing event enrichment analyses.

To plot individual isoform usage by differential gene expression, we combined the IsoformSwitchAnalyzeR dIF calculations and gene expression data from the aforementioned DESeq2 results. The top 30 isoforms per dataset comparison were identified by ranking isoforms by gene switch *q*-value, i.e., the significance of the summation of all isoform switching events per gene between mock and infected conditions.

A biological consequence is defined as the biological property of a transcript (i.e., domain region, ORF, etc.). After calculating the number of significant isoforms experiencing a biological consequence or alternative splicing event, we performed enrichment analysis to determine if a consequence or splicing event occurred more frequently in a particular direction (i.e., gain vs. loss) across conditions. For example, a fraction score of 0.5 implies that out of all significant isoforms experiencing consequence A, 50% experience a gain in consequence A and 50% experience a loss in consequence A, indicating no global preference in the direction the isoform population experiences consequence A. Statistical differences between consequence directions were calculated using a Fisher’s exact test and *p*-values were FDR adjusted.

### TE analysis

TE expression was quantified using the TEcount function from the TEtools software^118^. TEcount detects reads aligned against copies of each TE family annotated from the reference genome. DETEs in infected vs. mock conditions were detected using DEseq2 with a matrix of counts for genes and TE families as input. Functional enrichment of nearby genes (upstream 5 kb and downstream 1 kb of each TE copy within the human genome) was calculated with GREAT^51^ using options “genome background” and “basal + extension.” Only the occurrences that were identified as statistically significant by region using a binomial test were selected.

### Identification of putative binding sites for human RBPs on the SARS-CoV-2 genome

The list of RBPs downloaded from ATtRACT was filtered to retain only human RBPs. PWMs for these RBPs were obtained from ATtRACT. The PWM is a representation of the experimentally determined sequence-binding preferences of the RBP. These PWMs were further filtered to retain PWMs obtained through competitive experiments and to drop PWMs with very high entropy. This left 205 experimentally determined PWMs for 102 human RBPs. The SARS-CoV-2 reference genome sequence was scanned with these 205 PWMs to detect sequence matches using the TFBSTools R package (v1.20.0). This scored sub-sequences on the genome based on their sequence match to the given PWMs. A minimum score threshold of 90% was used to identify putative RBP-binding sites.

### Enrichment analysis for putative RBP-binding sites

The sequence of the SARS-CoV-2 genome was shuffled 1000 times. Each of the 1000 shuffled sequences was scanned for putative RBP-binding sites as described above. Next, the number of putative binding sites for each RBP was counted, and the mean and SD of the number of sites was calculated for each RBP across all 1000 shuffled sequences. The *z*-score for each RBP was then calculated as provided in Eq. (3):

3 $${Z}=\frac{{{\rm{S}}}_{{\rm{real}}}-{\bar{{\rm{S}}}}_{{\rm{shuffled}}}}{{{\rm{\sigma }}}_{{\rm{shuffled}}}}$$

where ${{\rm{S}}}_{{\rm{real}}}$ is the number of putative binding sites for the RBP on the real genome, ${\bar{{\rm{S}}}}_{{\rm{shuffled}}}$ is the mean number of putative binding sites for the RBP across 1000 shuffled sequences, and ${{\rm{\sigma }}}_{{\rm{shuffled}}}$ is the SD of the number of putative binding sites for the RBP across 1000 shuffled sequences. The two-tailed *p*-value for each RBP was calculated from the *z*-score. A minimum FDR-adjusted *p*-value of 0.01 was taken as the cutoff for significant enrichment or depletion.

The same analysis was repeated taking only the sequence of the 5′-UTR, 3′-UTR, or intergenic regions of the SARS-CoV-2 reference genome, and was also repeated using the negative-sense genome sequence. Finally, this analysis was repeated with the reference genomes of SARS-CoV and RaTG13.

### Conservation analysis for putative RBP-binding sites

The multiple sequence alignment of 181,003 SARS-CoV-2 genome sequences was downloaded from GISAID^119^. For each putative RBP-binding site, we selected the corresponding columns of the multiple sequence alignment. We then counted the number of genomes in which the sequence was identical to that of the reference genome.

### Viral genotype–phenotype association

All complete SARS-CoV-2 genomes having disease severity metadata in GISAID on 11 November 2020, together with the GenBank reference sequence, were aligned with MAFFT (v7.464) within a high-performance computing environment using 1 thread and the–nomemsave parameter^120^. Sequences responsible for introducing excessive gaps in this initial alignment were then manually identified and removed, leaving 8079 sequences that were then used to generate a new multiple sequence alignment.

The disease severity metadata for these sequences was then normalized into four categories: severe (862 samples), moderate (3873 samples), mild (2996 samples), and NA (310 samples). The complete correspondence between original patient status and these four categories can be found in Supplementary Data 17. These categories were based on whether the patient was treated in the intensive-care unit or died during acute infection, hospitalized or symptomatic, asymptomatic, not specified, or not available, respectively. The distribution of patient biological sex included males (4067 samples), females (3085 samples), unknown (798 samples), and not specified (129 samples). Patient age was converted into age ranges including the following: unspecified (38 samples), <20 (441 samples), 20–29 (1009 samples), 30–39 (1268 samples), 40–49 (1255 samples), 50–64 (1661), 65–74 (769), 75–84 (516 samples), >85 years old (283 samples), or not available (839 samples).

Next, the sequence data and associated metadata were used as input to the meta-CATS^59^ algorithm. Meta-CATS uses a *χ*^2^-statistical test to identify aligned positions containing significant differences in their base distribution between two or more metadata categories (e.g., severe vs. mild disease or male vs. female). The Benjamini–Hochberg multiple hypothesis correction was then applied to all positions^121^. Significant results were then evaluated against the annotated protein regions of the reference genome to determine their effect on amino acid sequence.

### Statistics and reproducibility

#### RNA-seq datasets

Biological replicates for individual conditions are described as follows: within GSE147507 series 1, 2, 5, 7, 8, and 9 consisted of three biological replicates; series 3 and 4 consisted of two biological replicates. Within GSE150316, Cases 8 and 9 along with the negative control consisted of five biological replicates; Cases 1 and 4 consisted of four biological replicates; Cases 2 and 11 consisted of three biological replicates; and Case 3 consisted of two biological replicates. More details on the samples and replicates for each dataset are given in Supplementary Data 1.

#### DEGs and TEs

Differential expression of genes and TEs was separately determined based on the counts of features with DESeq2 (v1.26.0)^103^, which is based on a negative binomial regression model. The method normalized sequencing depth using a median-to-ratio method; then a Bayesian shrinkage approach was used to estimate both coefficients and dispersion parameters in the negative binomial. Then, a Wald’s test was performed to identify DE genes or DETEs.

#### Enrichment analyses

The GO functional enrichment analysis was performed by retrieving all of the GO annotations for each DEG in each dataset. A hypergeometric statistical test was applied to all of the GO annotations for each DEG in each dataset, functions with an FDR-adjusted *p*-value < 0.05 were considered significantly overrepresented.

Genes with FDR-adjusted *p*-values < 0.05 based on DESeq2 were used as the gene list, whereas the superset of both significant and nonsignificant genes for each dataset was used as the background gene list. These lists of genes were then subjected to 5000 bootstrap replicates to generate a null distribution for each available pathway. Pathways that had a Bonferroni-adjusted *p*-value < 0.05 were labeled as statistically significant and were reported in the results.

#### Differential usage of isoforms

IsoformSwitchAnalyzeR R package (v1.11.3)^113^ detected genome-wide enrichment by counting isoform switches and comparing the number of gain vs. losses. Enrichment tests were performed via base R’s *prop.test* and comparisons of enrichments were done with *fisher.test*. FDR-adjusted *p*-values (*q*-values) inferior to 0.05 were considered significant.

#### Metabolic flux prediction

We ran EBSeq (v3.12)^112^ on the gene count matrix. EBSeq returned the posterior probabilities of each gene being differentially expressed (PPDE) and the log2 fold changes. Moomin^32^ then used the results of PPDE along with log2 fold changes to predict topological solutions within the metabolic network.

#### TE functional enrichment analysis

Based on DETEs, we used GREAT^51^ to analyze the functional significance of *cis*-regulatory regions. The method performs a binomial test on the total portion of the genome associated with any given ontology vs. the fraction of the input genomic regions which fall into those areas.

#### RBP analysis

Enrichment or depletion of putative RBP-binding sites in a sequence was calculated by shuffling the sequence 1000 times and scanning each shuffled sequence for putative RBP-binding sites. A *z*-score for each RBP was calculated as provided in Eq. (3):

$${Z}=\frac{{{\rm{S}}}_{{\rm{real}}}-{\bar{{\rm{S}}}}_{{\rm{shuffled}}}}{{{\rm{\sigma }}}_{{\rm{shuffled}}}}$$

where ${{\rm{S}}}_{{\rm{real}}}$ is the number of putative binding sites for the RBP on the real genome, ${\bar{{\rm{S}}}}_{{\rm{shuffled}}}$ is the mean number of putative binding sites for the RBP across 1000 shuffled sequences, and ${{\rm{\sigma }}}_{{\rm{shuffled}}}$ is the SD of the number of putative binding sites for the RBP across 1000 shuffled sequences. The two-tailed *p*-value for each RBP was calculated from the *z*-score. A minimum FDR-adjusted *p*-value of 0.01 was taken as the cutoff for significant enrichment or depletion.

#### Viral genotype–phenotype association

Over 8000 SARS-CoV-2 genomes that had associated clinical metadata such as disease severity, age, or biological sex were included in the analysis. The sequences were divided into categories based on the available clinical metadata before being subjected to a *χ*^2^-statistical test. Results that met an FDR-adjusted *p*-value < 0.05 were labeled as statistically significant and were manually reviewed to identify aligned positions that had the potential for statistical skew due to not surpassing the minimal number of bases in a given category (at least five viral strains having the same base in each category).

### Reporting summary

Further information on research design is available in the Nature Research Reporting Summary linked to this article.

## Supplementary information

Supplementary Information

Description of Additional Supplementary Files

Supplementary Data 1

Supplementary Data 2

Supplementary Data 3

Supplementary Data 4

Supplementary Data 5

Supplementary Data 6

Supplementary Data 7

Supplementary Data 8

Supplementary Data 9

Supplementary Data 10

Supplementary Data 11

Supplementary Data 12

Supplementary Data 13

Supplementary Data 14

Supplementary Data 15

Supplementary Data 16

Supplementary Data 17

Supplementary Data 18

Reporting Summary

## Data Availability

RNA-seq datasets with accessions GSE147507 and GSE150316 were obtained from the NCBI Gene Expression Omnibus (GEO) database (https://www.ncbi.nlm.nih.gov/geo). The reference genome sequences of SARS-CoV-2, RaTG13, and SARS-CoV were downloaded from Genbank under the accessions NC_045512, MN996532.1, and NC_004718.3, respectively. A list of known RNA-binding proteins (RBPs) and their Position Weight Matrices (PWMs) were downloaded from ATtRACT (https://attract.cnic.es/download). Normalized gene expression values in human lung tissue were obtained from the GTEx database, version 8 (https://gtexportal.org/home/datasets). Single-cell RNA-seq data for human lung cells were obtained from the NCBI GEO database under accession GSE122960. Finally, all SARS-CoV-2 complete genomes collected from humans, along with associated metadata, were downloaded from the GISAID database (https://www.gisaid.org/) on 11 November 2020^119^. Supplementary data have been deposited on Zenodo at 10.5281/zenodo.4644596^122^. Any other data are available from the corresponding authors on reasonable request.

## Code availability

Code for the analyses described in this work is available at https://github.com/vaguiarpulido/covid19-research^123^, under the MIT open-source license. The following versions of software were used in this study: STAR (v2.3.7a), Samtools (v1.10), StringTie (v2.1.1), DESeq2 (v1.26.0), GOstats package (v2.54.0), IsoformSwitchAnalyzeR R package (v1.11.3), Moomin (v1.0), EBSeq (v3.12), TETools (v1.0.0), sra-tools (v2.10.8), FastQC (v0.11.9), MultiQC (v1.9), TFBSTools R package (v1.20.0), and MAFFT (v7.464).
