# Supplementary material for: Genome-wide bioinformatic analyses predict key host and viral factors in SARS-CoV-2 pathogenesis
Source: Commun Biol. 2021 May 17;4:590. doi: 10.1038/s42003-021-02095-0 (PMC8128904; doi:10.1038/s42003-021-02095-0)
Supplement: Supplementary file 1 — Supplementary Information [file 42003_2021_2095_MOESM1_ESM.pdf]

Supplementary material for

Genome-wide bioinformatic analyses predict key host and viral factors in SARS-CoV-2 pathogenesis

Mariana G. Ferrarini[1*], Avantika Lal[2*], Rita Rebollo[1], Andreas J. Gruber[3], Andrea Guarracino[4], Itziar Martinez Gonzalez[5], Taylor Floyd[6], Daniel Siqueira de Oliveira[7], Justin Shanklin[8], Ethan Beausoleil[8], Taneli Pusa[9], Brett E. Pickett[8#], Vanessa Aguiar-Pulido[10#]

* These authors contributed equally
# Corresponding authors

[1] University of Lyon, INSA-Lyon, INRA, BF2I, Villeurbanne, France
[2] NVIDIA Corporation, Santa Clara, CA, USA
[3] Oxford Big Data Institute, Nuffield Department of Medicine, University of Oxford, Oxford, UK
[4] Centre for Molecular Bioinformatics, Department of Biology, University Of Rome Tor Vergata, Rome, Italy
[5] Amsterdam UMC, Amsterdam, The Netherlands
[6] Center for Neurogenetics, Weill Cornell Medicine, Cornell University, New York, NY, USA
[7] Laboratoire de Biométrie et Biologie Evolutive, Université de Lyon; Université Lyon 1; CNRS; UMR 5558, Villeurbanne, France
[8] Brigham Young University, Provo, UT, USA
[9] Luxembourg Centre for Systems Biomedicine, Belvaux, Luxembourg
[10] Department of Computer Science, University of Miami, Coral Gables, FL, USA

Emails for correspondence:
Brett E. Pickett: brett_pickett@byu.edu
Vanessa Aguiar-Pulido: vxa305@miami.edu

This document contains:

Supplementary Figures 1-6
Supplementary Tables 1-2

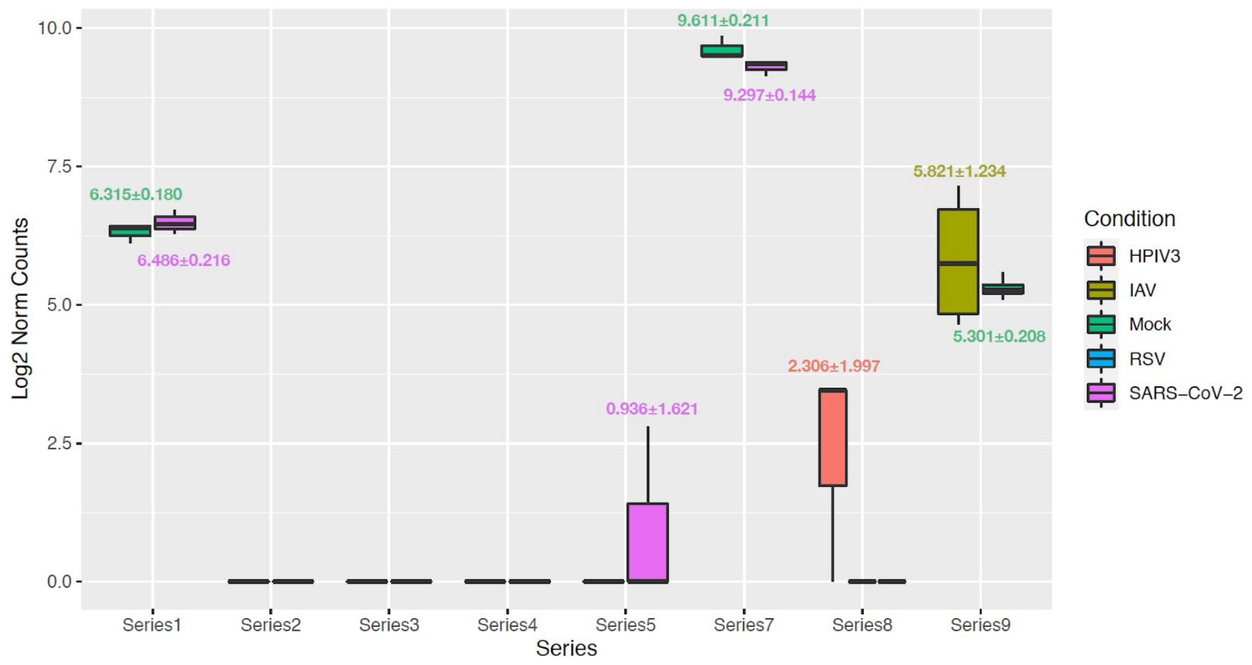

Supplementary Figure 1. Expression of the *ACE2* viral receptor gene in each RNA-seq dataset. The y-axis shows the log2 normalized counts for *ACE2* from DESeq2 results.

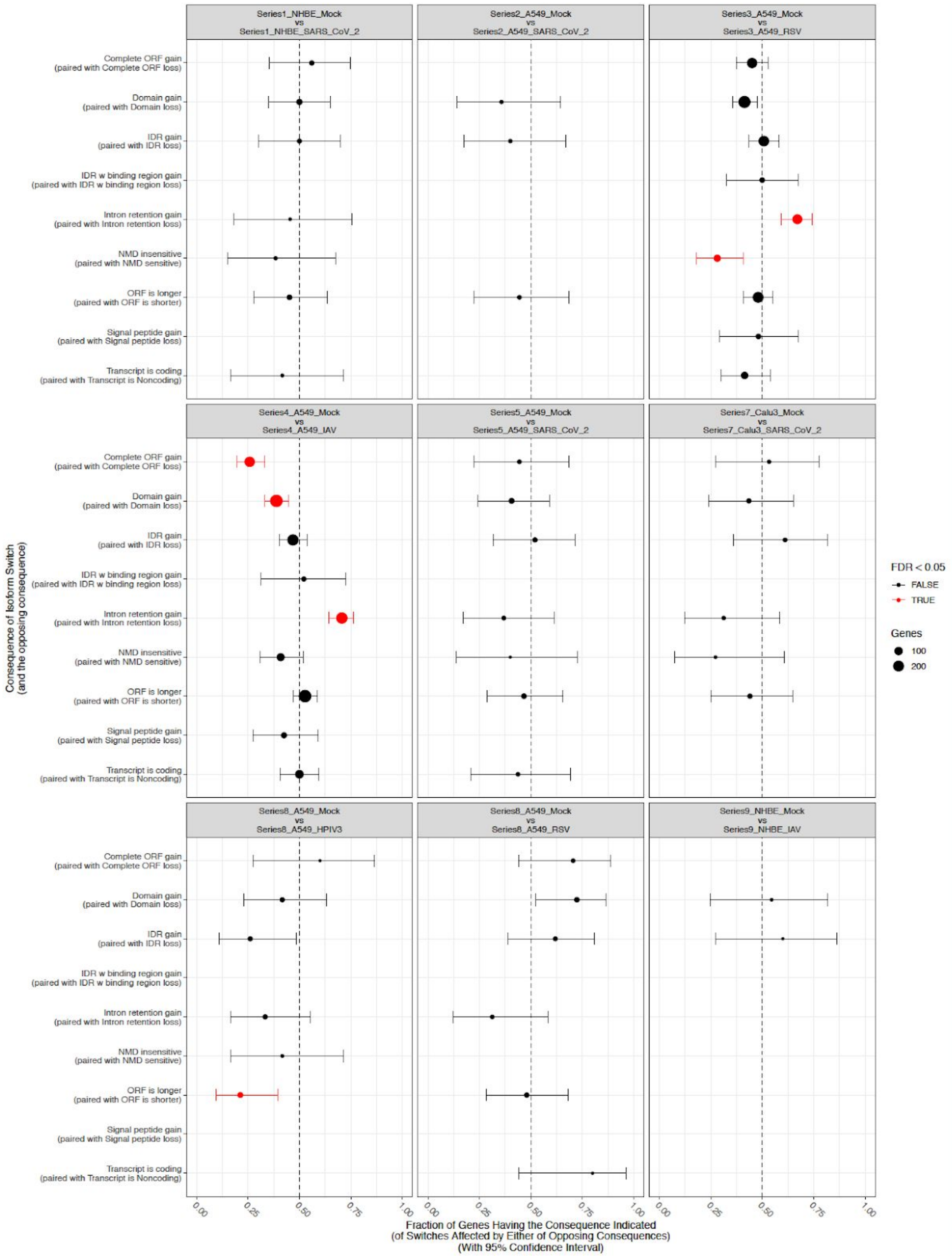

Supplementary Figure 2. Biological consequence enrichment analysis. A fraction of 0.5 on the x-axis (dashed line) suggests out of all significant isoforms experiencing consequence A (y-axis), 50% experience a gain in consequence A and 50% experience a loss in consequence A, indicating no global preference in the direction of consequence A for the isoform population. RSV, IAV, and HPIV3 infected samples exhibit significant global enrichment in consequences shortening the ORF, increasing sensitivity to NMD, and higher IR rates. Error bars represent 95% confidence intervals. Red error bars = significant consequence enrichment (adjusted p-value < 0.05), black error bars = non-significant consequence enrichment. ORF = open reading frame, IDR = intrinsically disordered region, NMD = nonsense-mediated decay.

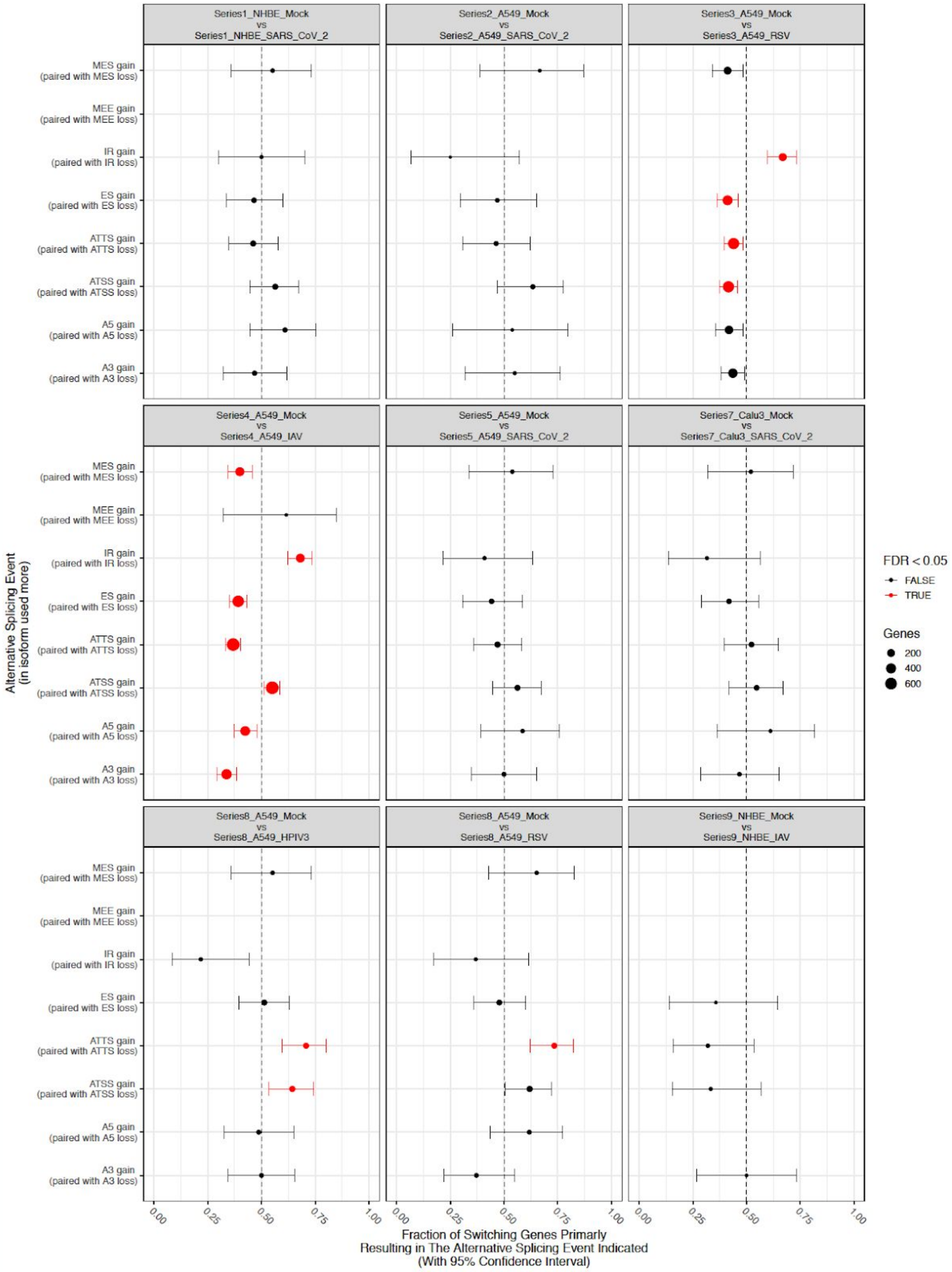

Supplementary Figure 3. Enrichment analysis for alternative splicing events. RSV, IAV, and HPIV3 infected samples display global enrichment of increased IR and ATSS, alongside decreased exon skipping (MES and ES), alternative splice site usage (A5 and A3), and ATTS. Error bars represent 95% confidence intervals. Red error bars = significant consequence enrichment (p-value < 0.05), black error bars = non-significant consequence enrichment. MES = multiple exon skipping, MEE = mutually exclusive exons, IR = intron retention, ES = exon skipping, ATTS = alternative transcription termination site, ATSS = alternative transcription start site, A5 = alternative 5' splice site, A3 = alternative 3' splice site.

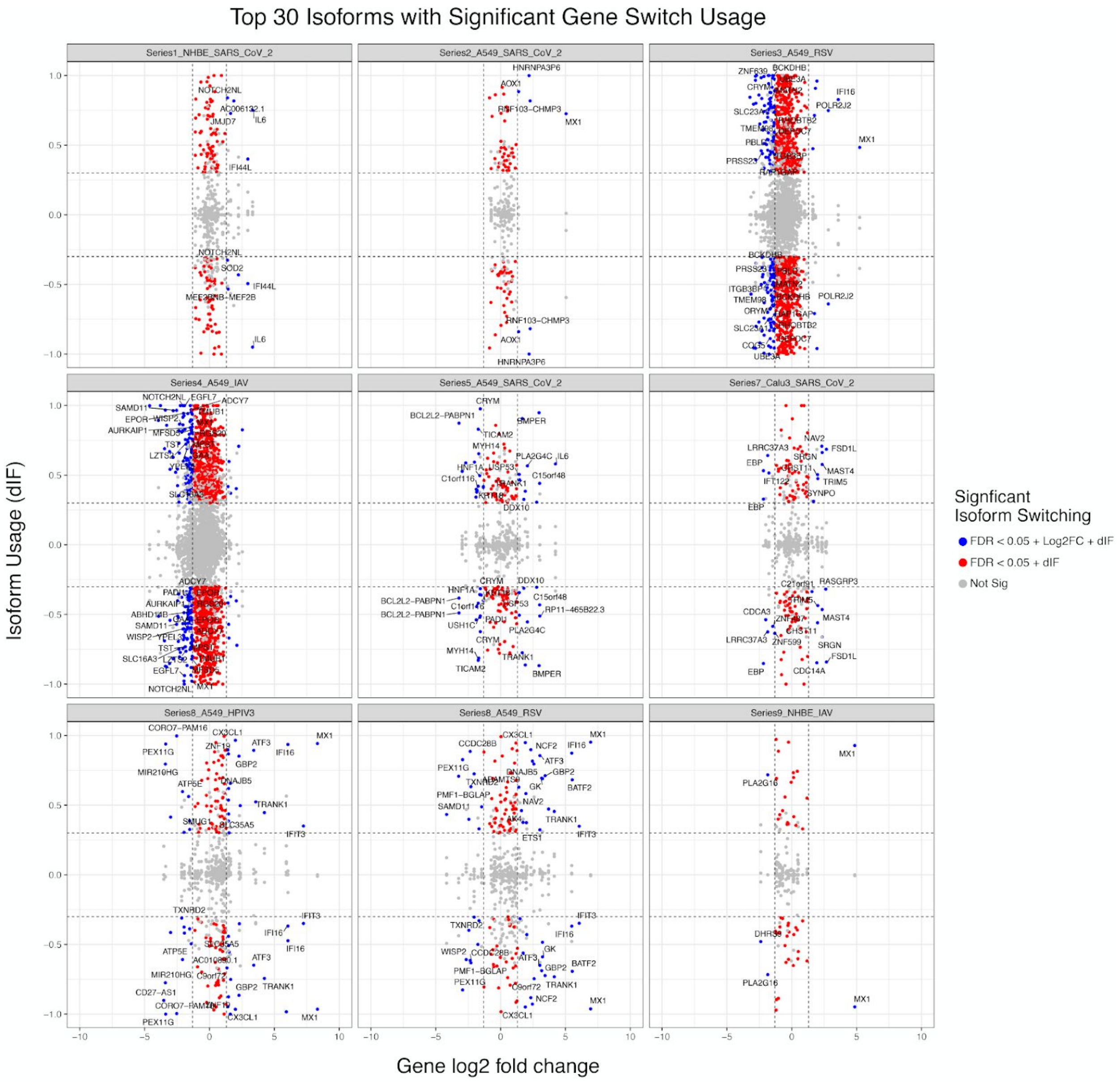

Supplementary Figure 4. Top 30 isoforms experiencing significant isoform usage and differential gene expression. Blue circles = isoforms experiencing significant switch in usage (dIF ≥ |0.3|), gene log2 fold change ≥ |1|, and adjusted p-value < 0.05; red circles = isoforms experiencing significant switch in usage (dIF ≥ |0.3|) and adjusted p-value < 0.05; gray circles = non-significant isoforms. Horizontal dotted lines = dIF ≥ |0.3|, vertical dotted lines = gene log2 fold change ≥ |1|.

A

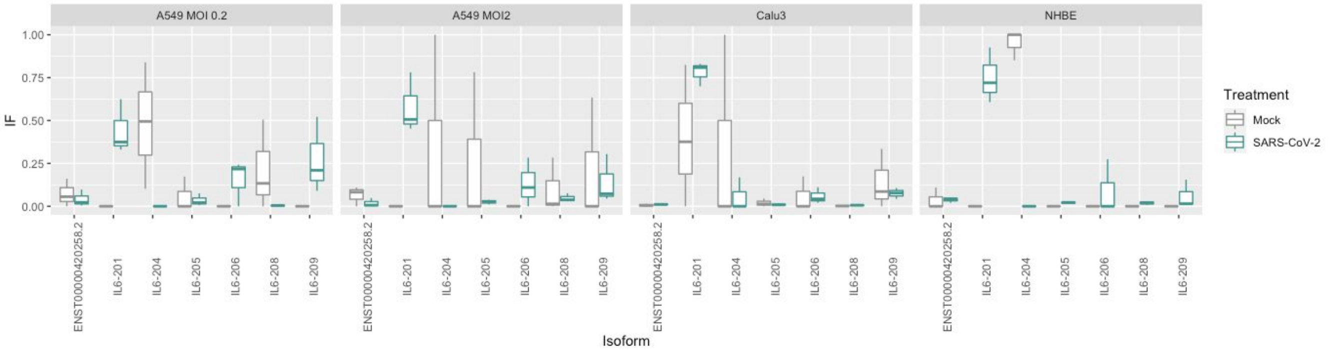

B

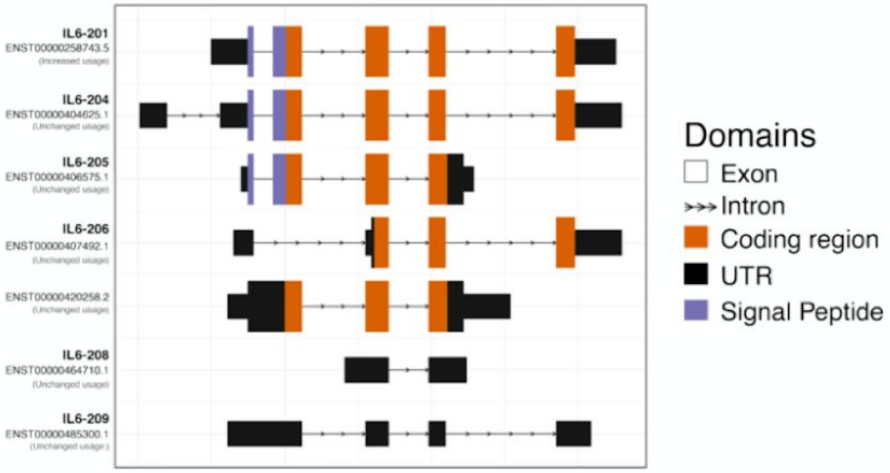

Supplementary Figure 5. Isoform usage analyses specifically with *IL6* transcripts from IsoformSwitchAnalyzeR software. A) Isoform fractions of the 7 detected *IL-6* isoforms within the 4 datasets of cell lines infected with SARS-CoV-2. Boxplots represent biological triplicates of each condition B) Isoform splice graphs for the detected isoforms of *IL-6*. MOI: Multiplicity of Infection.

A

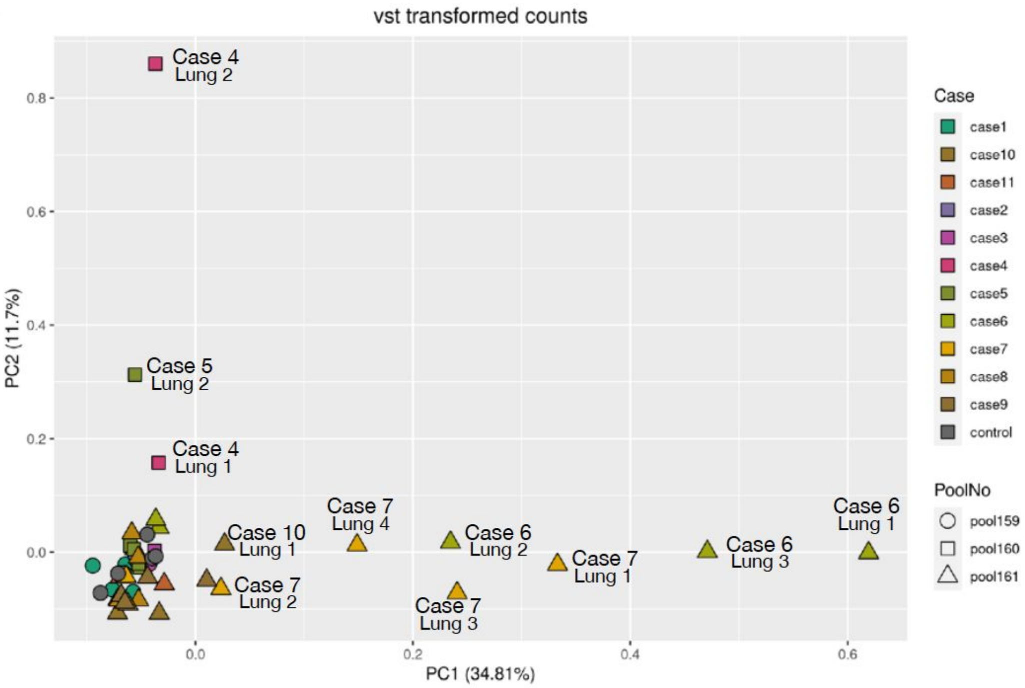

B

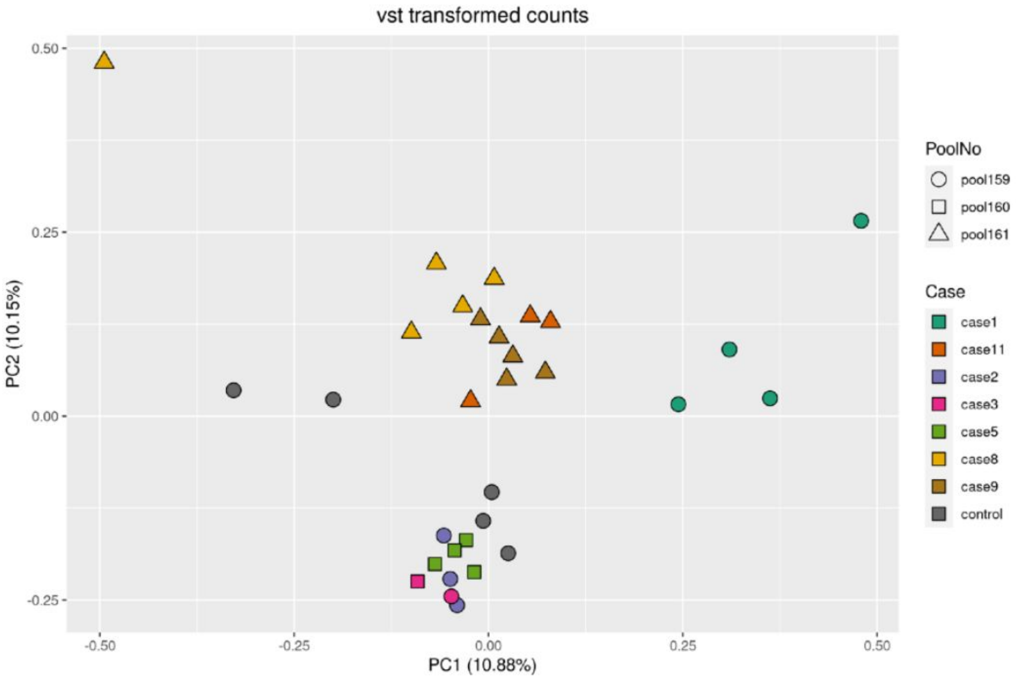

Supplementary Figure 6. PCA plots for samples from the dataset GSE150316. A) PCA was performed based on the transformed values obtained after applying the variance stabilizing transformation [28] implemented in the vst() function of DESeq2 [27]. Four outlier samples (Cases 4, 6, 7 and 10) were identified and discarded. B) PCA was repeated after discarding these samples. PCA: Principal Component Analysis.

Comparison	Number of significant isoforms	Number of genes
Series1 NHBE Mock vs Series1 NHBE SARS CoV 2	145	101
Series2 A549 Mock vs Series2 A549 SARS CoV 2	82	54
Series3 A549 Mock vs Series3 A549 RSV	1281	710
Series4 A549 Mock vs Series4 A549 IAV	1690	956
Series5 A549 Mock vs Series5 A549 SARS CoV 2	172	105
Series7 Calu3 Mock vs Series7 Calu3 SARS CoV 2	133	86
Series8 A549 Mock vs Series8 A549 HPIV3	165	109
Series8 A549 Mock vs Series8 A549 RSV	148	101
Series9 NHBE Mock vs Series9 NHBE IAV	48	32
Combined	3569	1960

Supplementary Table 1. Isoform analysis summary. Summary results for the number of significant isoforms and corresponding genes quantified. Significant isoforms were identified by exhibiting a change in usage greater than or equal to 30% in absolute value (dIF $\geq |0.3|$) and an adjusted p-value of 0.05.

ID	start	end	strand	sequence	RBP	region	Genomes	Fraction of genomes
NC_045512.2	85	91	+	TGTGTGG	CELF5, RBM24	5’ UTR	173442	0.9582272117
NC_045512.2	154	160	+	TGACAGG	FMR1	5’ UTR	175669	0.9705308752
NC_045512.2	159	165	+	GGACACG	FMR1	5’ UTR	175713	0.9707739651
NC_045512.2	235	240	+	AGGTTT	ZRANB2	5’ UTR	176643	0.9759120015
NC_045512.2	247	253	+	GGTGTGA	RBM24	5’ UTR	175869	0.9716358292
NC_045512.2	259	264	+	AGGTAA	ZRANB2	5’ UTR	176118	0.973011497
NC_045512.2	21552	21557	+	CTAAAC	KHDRBS1	intergenic	179409	0.9911935161
NC_045512.2	21556	21562	+	ACGAACA	PABPC1	intergenic	179423	0.9912708629
NC_045512.2	26469	26474	+	CTAAAC	KHDRBS1	intergenic	180659	0.998099479
NC_045512.2	26478	26483	+	CTAAAT	KHDRBS1	intergenic	180688	0.9982596974
NC_045512.2	26479	26484	+	TAAATA	PPIE	intergenic	180683	0.9982320735
NC_045512.2	26480	26485	+	AAATAT	PPIE	intergenic	180625	0.9979116368
NC_045512.2	26481	26486	+	AATATT	PPIE	intergenic	180611	0.99783429
NC_045512.2	26482	26487	+	ATATTA	PPIE	intergenic	180601	0.9977790423
NC_045512.2	26483	26488	+	TATTAT	PPIE	intergenic	180601	0.9977790423
NC_045512.2	26484	26489	+	ATTATA	PPIE	intergenic	180601	0.9977790423
NC_045512.2	26484	26487	+	ATTA	TIAL1	intergenic	180615	0.9978563891
NC_045512.2	26485	26490	+	TTATAT	PPIE	intergenic	180596	0.9977514185
NC_045512.2	26486	26491	+	TATATT	PPIE	intergenic	180568	0.9975967249
NC_045512.2	26487	26492	+	ATATTA	PPIE	intergenic	180632	0.9979503102
NC_045512.2	26489	26492	+	ATTA	TIAL1	intergenic	180649	0.9980442313
NC_045512.2	26490	26496	+	TTAGTTT	ELAVL1	intergenic	180580	0.9976630222
NC_045512.2	26493	26498	+	GTTTTT	TIA1, ELAVL2, CELF2	intergenic	180553	0.9975138534
NC_045512.2	26493	26499	+	GTTTTTC	TIA1, ELAVL2, TIAL1	intergenic	180514	0.9972983873
NC_045512.2	26494	26499	+	TTTTTC	TIA1, ELAVL2, TIAL1	intergenic	180532	0.9973978332
NC_045512.2	26494	26498	+	TTTTT	CELF2	intergenic	180571	0.9976132992
NC_045512.2	26500	26504	+	TGTTT	CELF2	intergenic	180495	0.9971934167
NC_045512.2	26501	26505	+	GTTTG	TIAL1, TIA1	intergenic	180506	0.9972541892
NC_045512.2	26502	26508	+	TTTGGAA	EIF4B	intergenic	180517	0.9973149616
NC_045512.2	26509	26513	+	CTTTA	ELAVL2, TIAL1, TIA1	intergenic	180625	0.9979116368
NC_045512.2	26510	26515	+	TTTAAT	PPIE	intergenic	180639	0.9979889836
NC_045512.2	26511	26516	+	TTAATT	PPIE	intergenic	180645	0.9980221322
NC_045512.2	26512	26517	+	TAATTT	PPIE	intergenic	180655	0.9980773799
NC_045512.2	26513	26518	+	AATTTT	PPIE	intergenic	180677	0.9981989249
NC_045512.2	26514	26519	+	ATTTTA	PPIE, TIA1, ELAVL2, TIAL1	intergenic	180694	0.998292846
NC_045512.2	27383	27388	+	ATTAAA	PPIE	intergenic	179804	0.9933758004
NC_045512.2	27384	27389	+	TTAAAC	KHDRBS1	intergenic	179742	0.9930332646
NC_045512.2	27388	27394	+	ACGAACA	PABPC1	intergenic	180525	0.9973591598
NC_045512.2	27884	27889	+	CTAAAC	KHDRBS1	intergenic	179777	0.9932266316
NC_045512.2	27888	27894	+	ACGAACA	PABPC1	intergenic	179769	0.9931824334
NC_045512.2	28256	28261	+	CTAAAC	KHDRBS1	intergenic	180576	0.9976409231
NC_045512.2	28260	28266	+	ACGAACA	PABPC1	intergenic	180869	0.9992596808
NC_045512.2	28269	28274	+	CTAAAA	KHDRBS1	intergenic	180816	0.998966868
NC_045512.2	28270	28275	+	TAAAAT	PPIE	intergenic	180883	0.9993370276
NC_045512.2	29530	29535	+	CTAAAC	KHDRBS1	intergenic	180028	0.994613349
NC_045512.2	29680	29685	+	TTTAAT	PPIE	3’UTR	177005	0.9779119683
NC_045512.2	29698	29704	+	TTAGGGA	HNRNPA1, HNRNPA1L2, HNRNPA2B1	3’UTR	174955	0.9665861892
NC_045512.2	29699	29704	+	TAGGGA	HNRNPA1	3’UTR	174974	0.9666911598
NC_045512.2	29701	29707	+	GGGAGGA	LIN28A	3’UTR	174934	0.966470169
NC_045512.2	29743	29749	+	CGGAGTA	LIN28A	3’UTR	172413	0.9525422231

Supplementary Table 2. Conservation of binding motifs for human RBPs across genome sequences of SARS-CoV-2 isolates. Conserved binding sites are listed along with the number and fraction of genomes in which the sequence is conserved. RBP: RNA Binding Protein.
