# Supplementary material for: Genome-wide bioinformatic analyses predict key host and viral factors in SARS-CoV-2 pathogenesis
Source: Commun Biol. 2021 May 17;4:590. doi: 10.1038/s42003-021-02095-0 (PMC8128904; doi:10.1038/s42003-021-02095-0)
Supplement: Supplementary file 2 — Description of Additional Supplementary Files [file 42003_2021_2095_MOESM2_ESM.pdf]

Description of Additional Supplementary Files

File name: Supplementary Data 1

Description: List of RNA-seq samples. Description and availability of the RNA-seq samples used in our study.

File name: Supplementary Data 2

Description: Additional lists of differentially expressed genes (DEGs):

a) List of 73 selected DEGs consistently and specifically altered in SARS-CoV-2 infection.

b) DEGs present in Cell Lines infected with SARS-CoV-2 and not detected in cell lines infected with IAV, RSV and HPIV3.

c) DEGs detected in all datasets including lung biopsies.

File name: Supplementary Data 3

Description: Results of differential gene expression analysis. Zipped file containing the complete results of differential gene expression analysis for all samples.

File name: Supplementary Data 4

Description: GO term enrichment results. Zipped file containing the results of GO term enrichment for all samples.

File name: Supplementary Data 5

Description: Results of functional enrichment analyses specific to SARS-CoV-2 infection:

a) GO enrichment analysis for DEGs specific in SARS-CoV-2 datasets.

b) Common and unique GO terms across all viral datasets analysed.

c) Detailed information for GO terms specific to each cell line infected with SARS-CoV-2.

File name: Supplementary Data 6

Description: Results of pathway enrichment for each dataset. Results of SPIA and DAVID analyses merged into one file.

File name: Supplementary Data 7

Description: Metabolic flux changes predicted for each dataset using Moomin.

File name: Supplementary Data 8

Description: Results of differential isoform analyses.

a) Differential gene expression, isoform expression and isoform usage data between conditions.

b) Output of all transcript features analyzed for the biological consequence enrichment analysis between conditions.

c) Occurrences of alternative splicing events for each transcript.

d) Protein domain types, locations and significance scoring.

e) Quantification and categorization for intrinsically disordered regions classification.

f) Changes in the presence and positioning of open reading frames across transcripts.

File name: Supplementary Data 9

Description: Transposable Element (TE) expression analysis. Zipped file containing the results of read count quantification and differential expression analysis for TE families in all cell line samples.

File name: Supplementary Data 10

Description: Functional enrichment of genes near Differentially Expressed Transposable Elements (DETEs). Zipped file containing the results of GREAT functional enrichment analysis (complete and per family) for all cell line samples.

File name: Supplementary Data 11

Description: Putative binding sites for human RBPs on the SARS-CoV-2 genome.

File name: Supplementary Data 12

Description: RNA Binding Protein (RBP) motif enrichment in SARS-CoV-2. Enrichment or depletion of binding motifs for human RBPs on the SARS-CoV-2 genome.

File name: Supplementary Data 13

Description: Biological evidence associated with candidate RBPs. Biological evidence from various sources, associated with the putative SARS-CoV-2 interacting human RBPs.

File name: Supplementary Data 14

Description: RNA Binding Protein (RBP) motif enrichment in SARS-CoV. Enrichment or depletion of binding motifs for human RBPs on the SARS-CoV genome.

File name: Supplementary Data 15

Description: RNA Binding Protein (RBP) motif enrichment in RaTG13. Enrichment or depletion of binding motifs for human RBPs on the RaTG13 genome.

File name: Supplementary Data 16

Description: Association of viral variants with patient information. Zipped file containing results of Chi-squared analyses to associate viral genome sequence variants with patient age range, biological sex, and disease severity.

File name: Supplementary Data 17

Description: Categorization of patients by disease status. Normalization of patient status into four categories: mild, moderate, severe and NA.

File name: Supplementary Data 18

Description: Supporting information for Figures 2 and 3. Data necessary to replicate figures 2b, 2c, 2d, 3b, and 3c.
