# Supplementary figures and images for: Genome-wide bioinformatic analyses predict key host and viral factors in SARS-CoV-2 pathogenesis
Source: Commun Biol. 2021 May 17;4:590. doi: 10.1038/s42003-021-02095-0 (PMC8128904; doi:10.1038/s42003-021-02095-0)

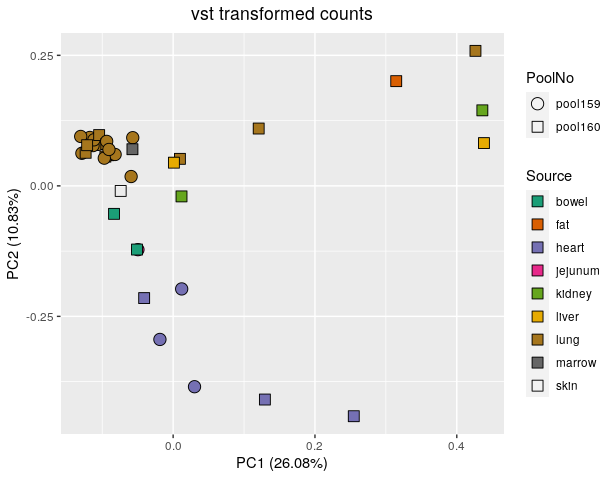

Supplement: Supplementary file 5 — Supplementary Data 3 [file 42003_2021_2095_MOESM5_ESM.zip › GSE150316_ValidationDataset/PCA_SourcePoolNo.png]

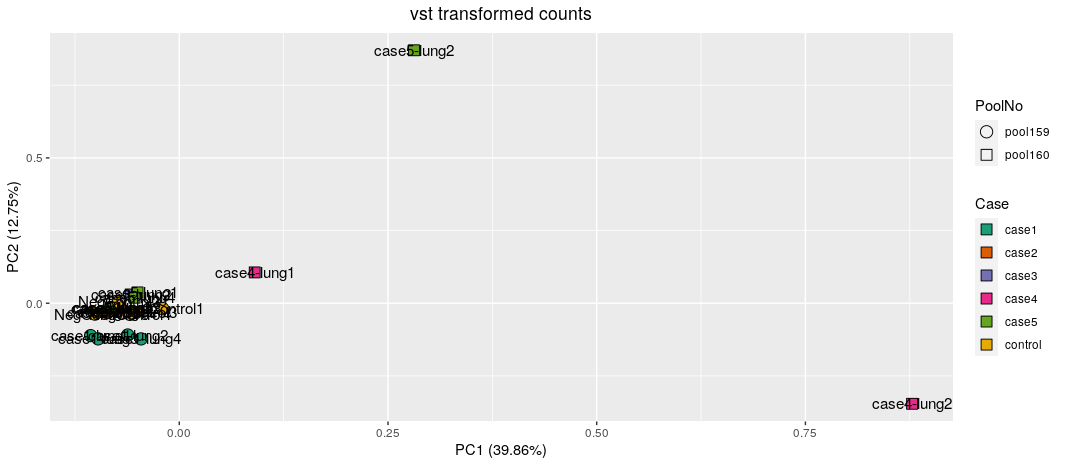

Supplement: Supplementary file 5 — Supplementary Data 3 [file 42003_2021_2095_MOESM5_ESM.zip › GSE150316_ValidationDataset/PCA_SourcePoolNo_Lung_WithLabels.png]

**Lung samples**  
**vst norm – pearson correlation**

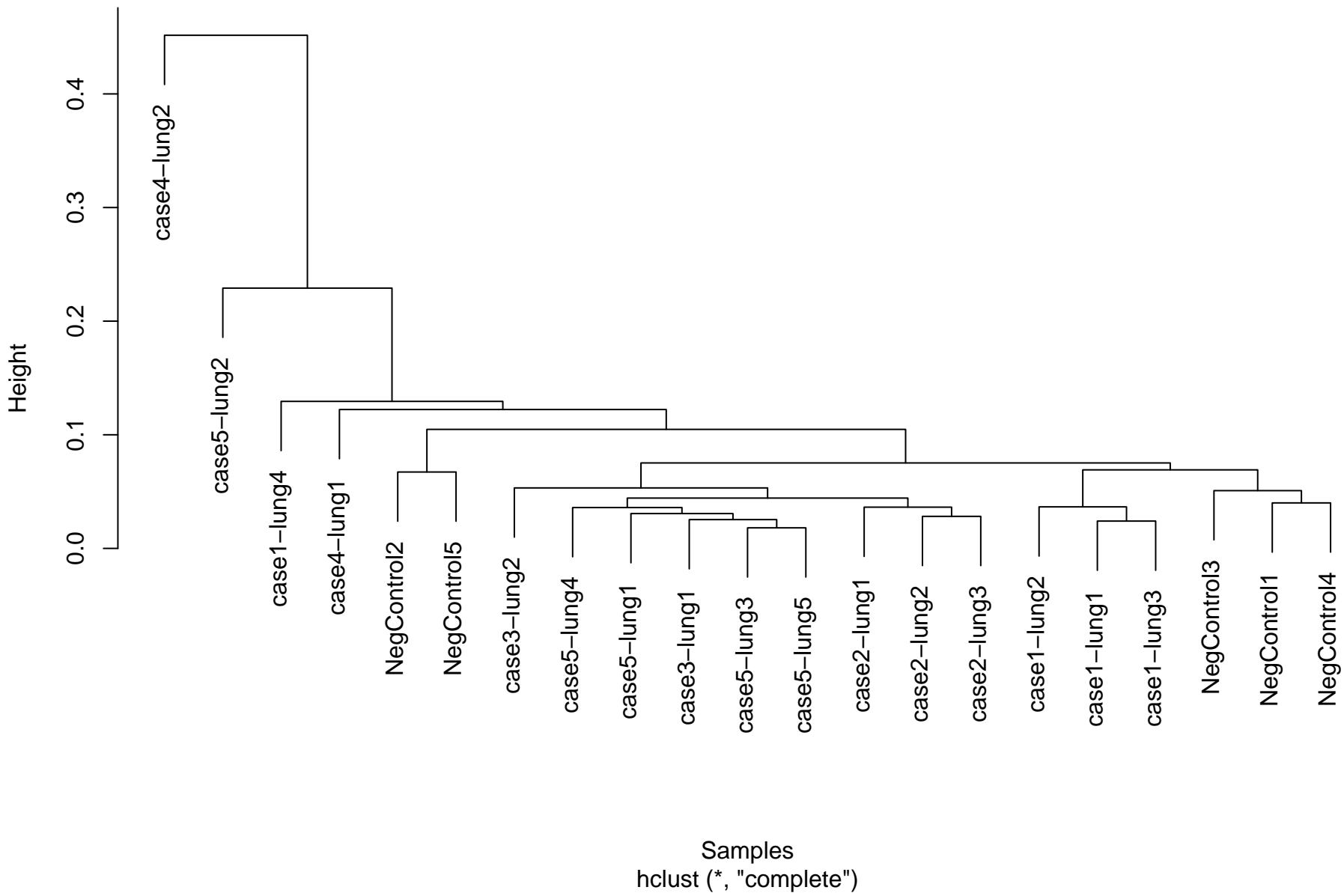

Supplement: Supplementary file 5 — Supplementary Data 3 [file 42003_2021_2095_MOESM5_ESM.zip › GSE150316_ValidationDataset/HierarchicalClustering/LungSamples_HierarchicalClustering_PearsonCorrelation.pdf]

**Lung samples**  
**vst norm – spearman correlation**

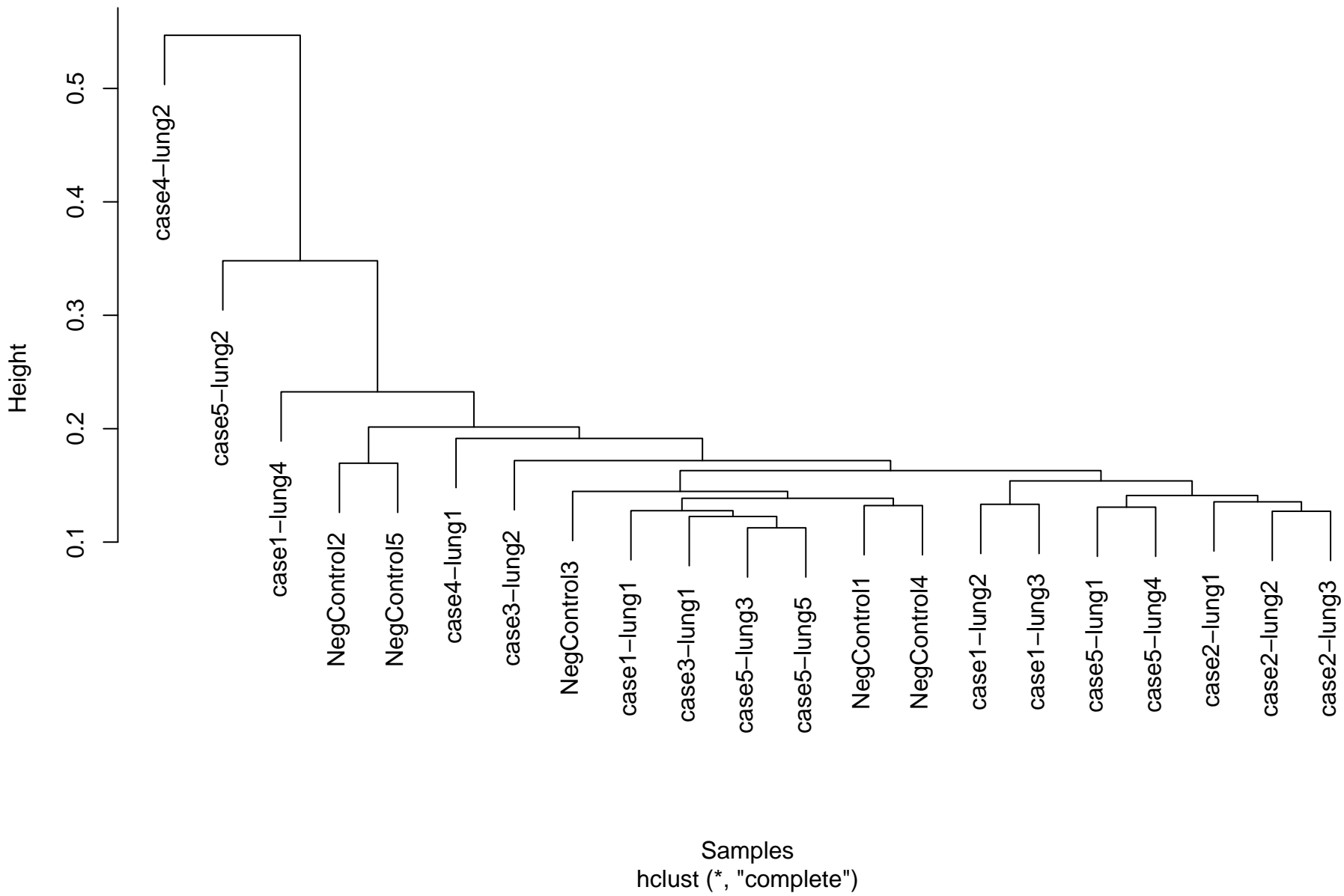

Supplement: Supplementary file 5 — Supplementary Data 3 [file 42003_2021_2095_MOESM5_ESM.zip › GSE150316_ValidationDataset/HierarchicalClustering/LungSamples_HierarchicalClustering_SpearmanCorrelation.pdf]

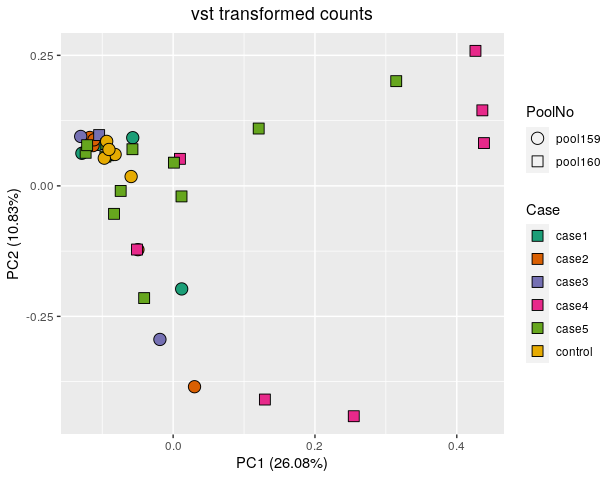

Supplement: Supplementary file 5 — Supplementary Data 3 [file 42003_2021_2095_MOESM5_ESM.zip › GSE150316_ValidationDataset/PCA_CasePoolNo.png]

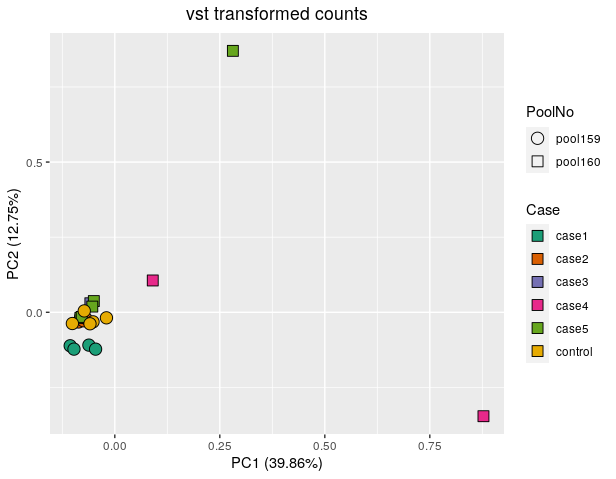

Supplement: Supplementary file 5 — Supplementary Data 3 [file 42003_2021_2095_MOESM5_ESM.zip › GSE150316_ValidationDataset/PCA_SourcePoolNo_Lung.png]

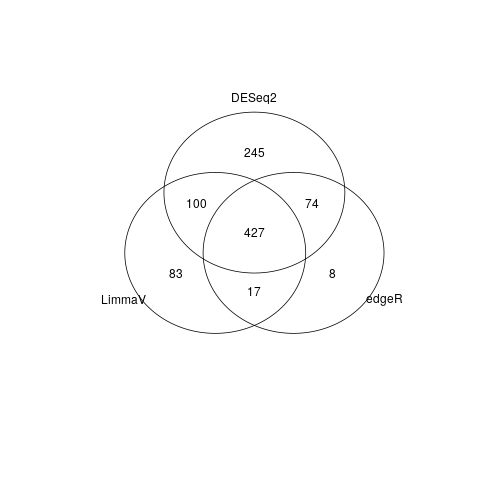

Supplement: Supplementary file 5 — Supplementary Data 3 [file 42003_2021_2095_MOESM5_ESM.zip › GSE150316_ValidationDataset/WithoutThreeOutliers/case1/des_Case/controlVscase1_edgeR-DESeq2-limmaVoom_venn.png]

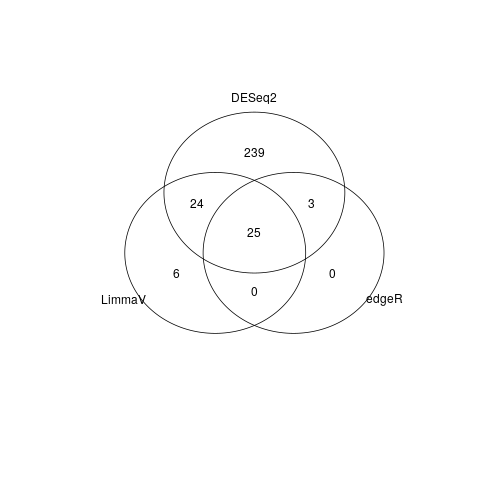

Supplement: Supplementary file 5 — Supplementary Data 3 [file 42003_2021_2095_MOESM5_ESM.zip › GSE150316_ValidationDataset/WithoutThreeOutliers/case5/des_Case/controlVscase5_edgeR-DESeq2-limmaVoom_venn.png]

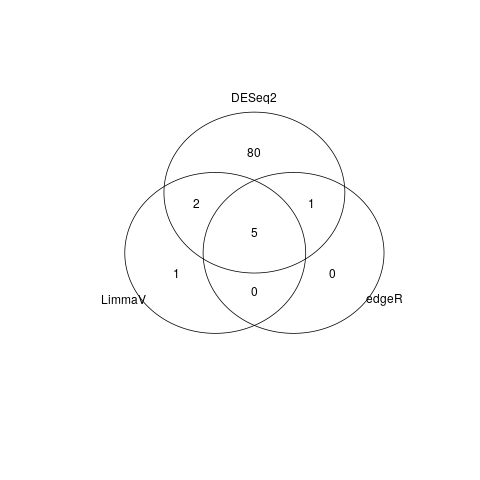

Supplement: Supplementary file 5 — Supplementary Data 3 [file 42003_2021_2095_MOESM5_ESM.zip › GSE150316_ValidationDataset/WithoutThreeOutliers/case2/des_Case/controlVscase2_edgeR-DESeq2-limmaVoom_venn.png]

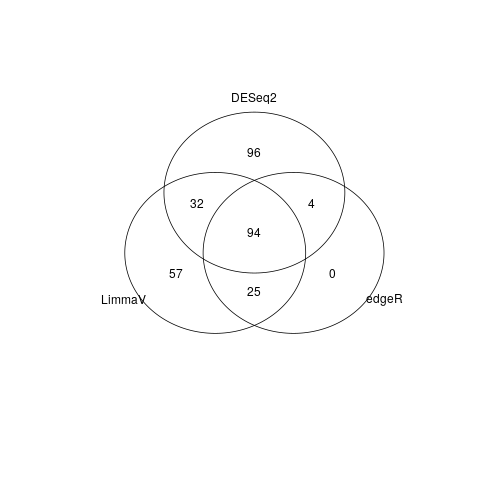

Supplement: Supplementary file 5 — Supplementary Data 3 [file 42003_2021_2095_MOESM5_ESM.zip › GSE150316_ValidationDataset/WithoutThreeOutliers/case3/des_Case/controlVscase3_edgeR-DESeq2-limmaVoom_venn.png]

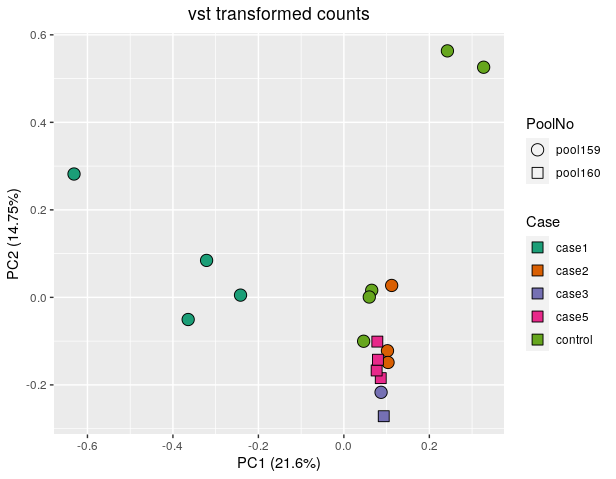

Supplement: Supplementary file 5 — Supplementary Data 3 [file 42003_2021_2095_MOESM5_ESM.zip › GSE150316_ValidationDataset/PCA_SourcePoolNo_Lung_NoOutliers.png]

# Lung samples

## vst norm – euclidian distance

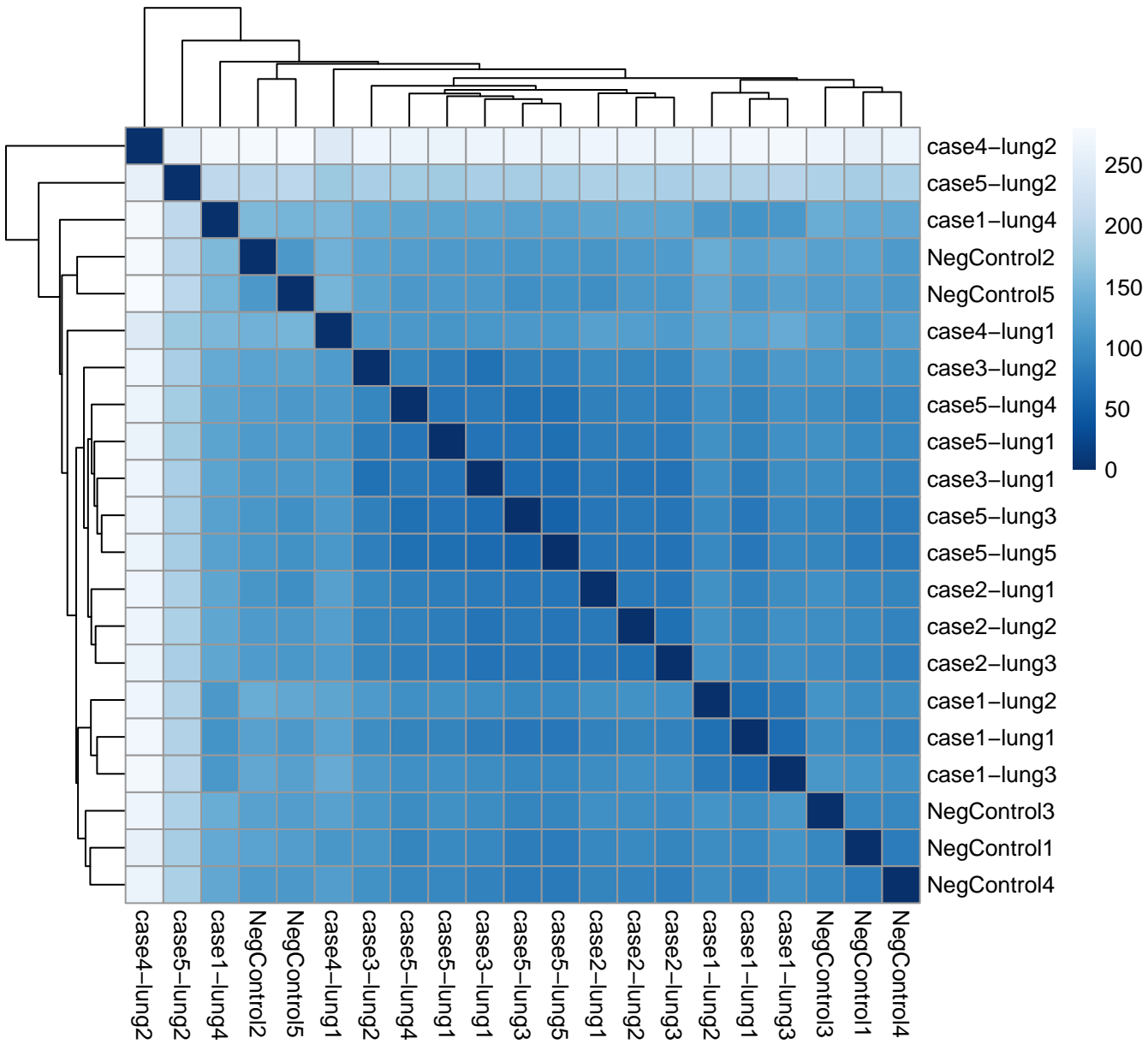

Supplement: Supplementary file 5 — Supplementary Data 3 [file 42003_2021_2095_MOESM5_ESM.zip › GSE150316_ValidationDataset/Heatmaps/LungSamples_EuclideanDistance.pdf]

# Lung samples

## vst norm – spearman correlation

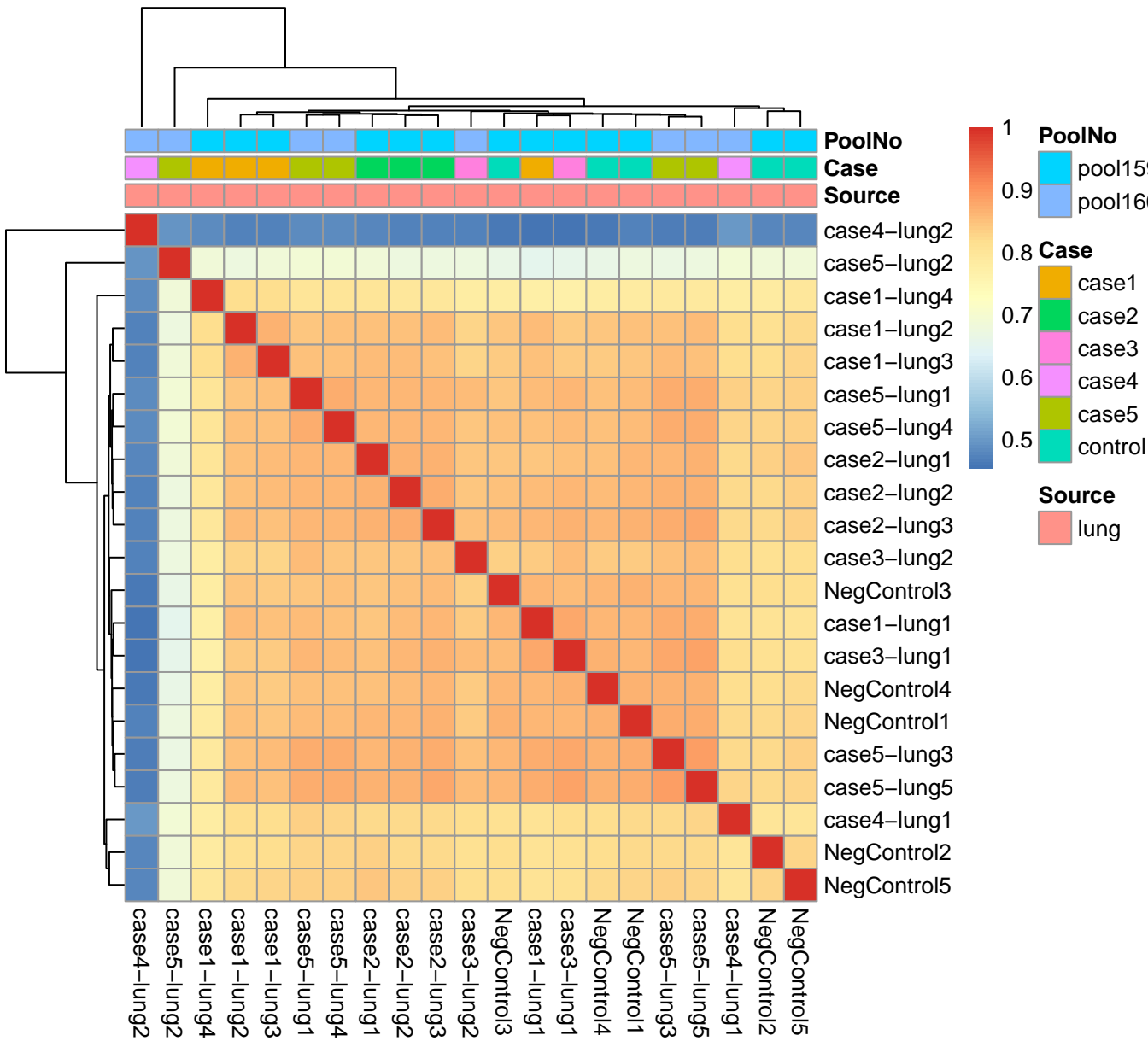

Supplement: Supplementary file 5 — Supplementary Data 3 [file 42003_2021_2095_MOESM5_ESM.zip › GSE150316_ValidationDataset/Heatmaps/LungSamples_SpearmanCorrelation.pdf]

# Lung samples

## vst norm – pearson correlation

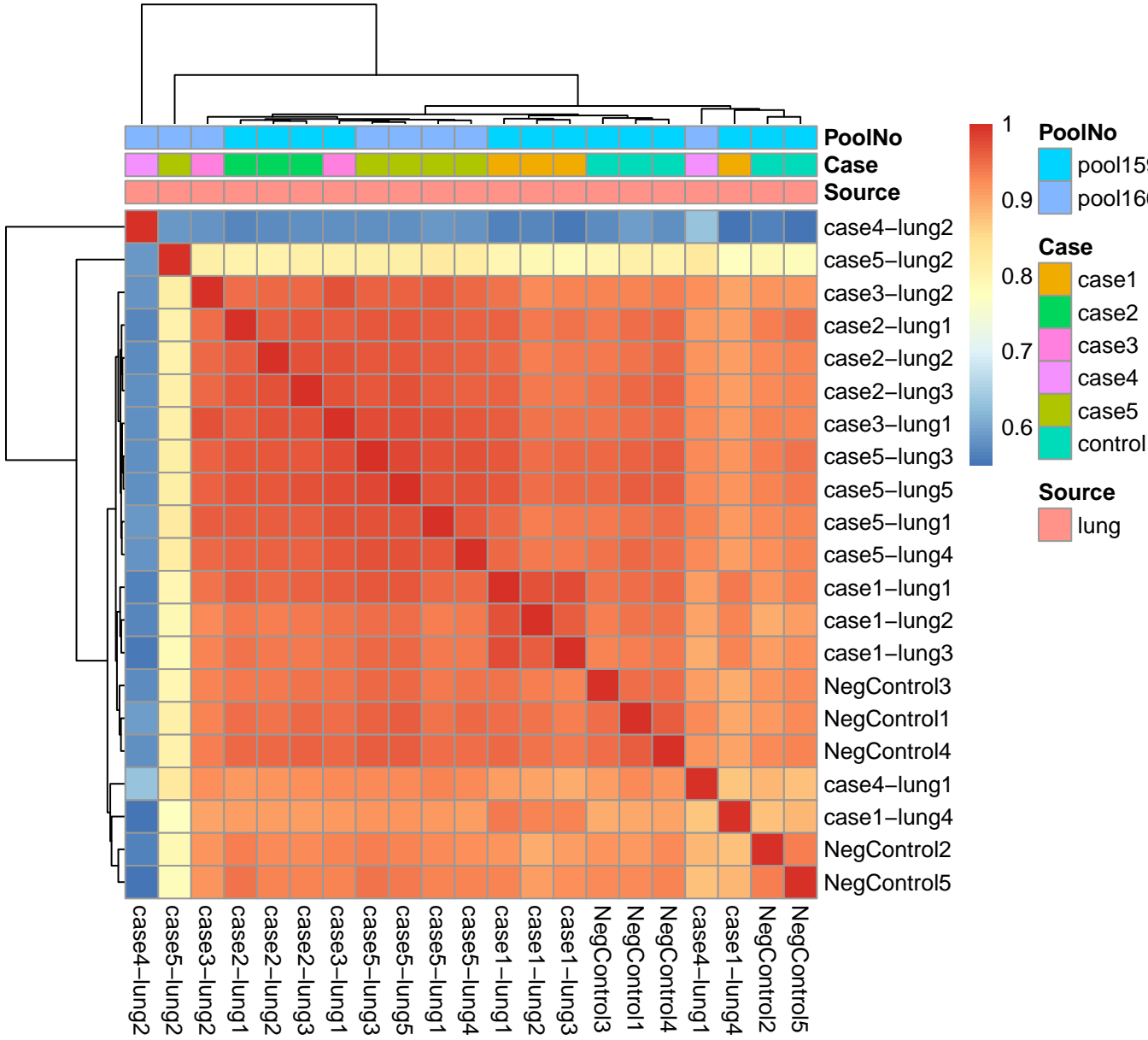

Supplement: Supplementary file 5 — Supplementary Data 3 [file 42003_2021_2095_MOESM5_ESM.zip › GSE150316_ValidationDataset/Heatmaps/LungSamples_PearsonCorrelation.pdf]

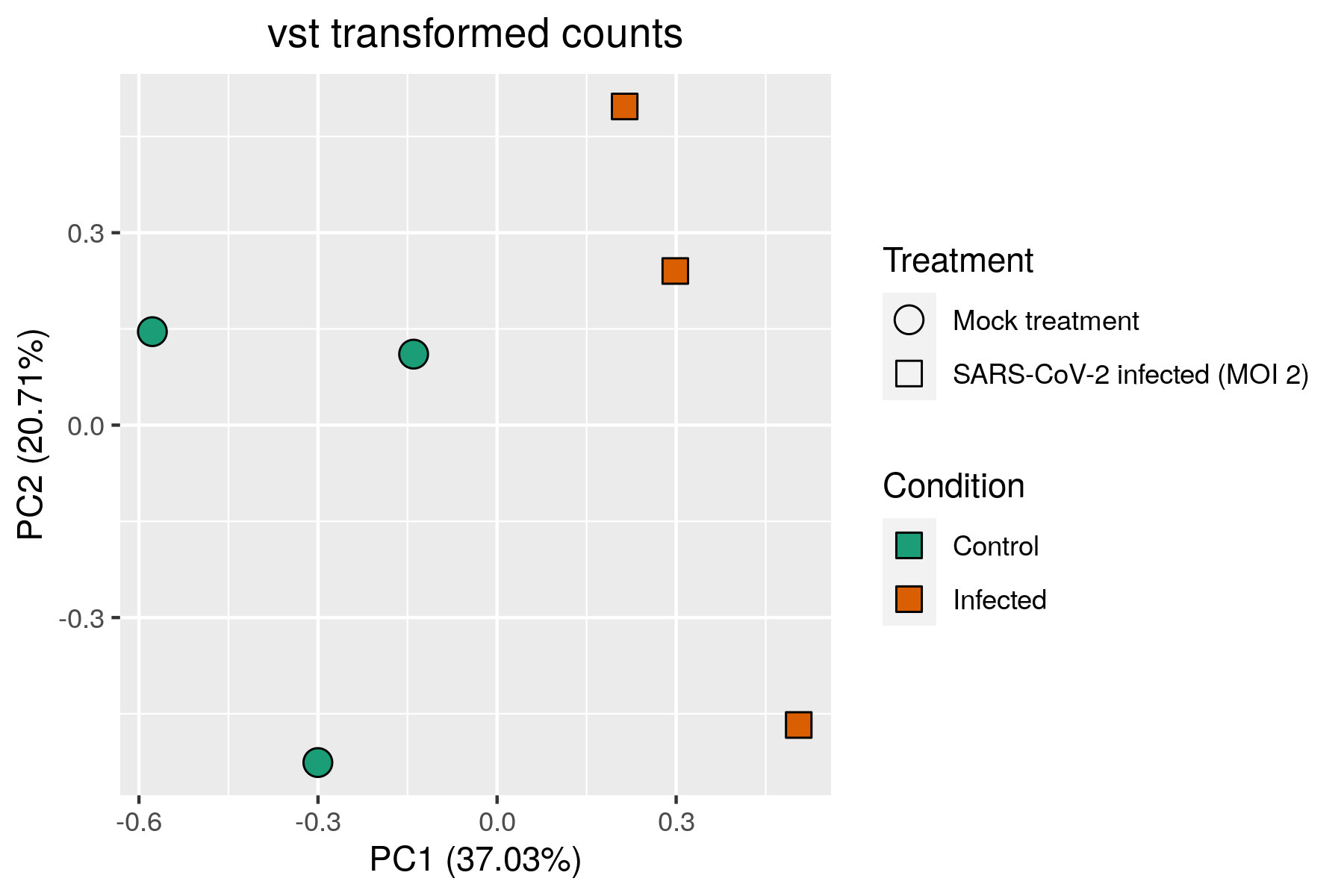

Supplement: Supplementary file 5 — Supplementary Data 3 [file 42003_2021_2095_MOESM5_ESM.zip › GSE147507_MainDataset/Series1_SARS-CoV-2/PCA_ConditionTreatment.png]

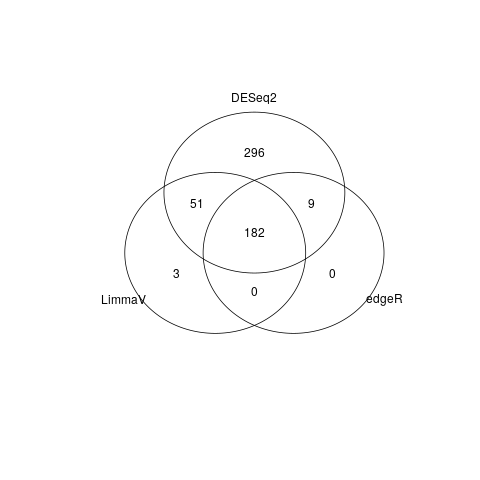

Supplement: Supplementary file 5 — Supplementary Data 3 [file 42003_2021_2095_MOESM5_ESM.zip › GSE147507_MainDataset/Series1_SARS-CoV-2/des_Condition/ControlVsInfected_edgeR-DESeq2-limmaVoom_venn.png]

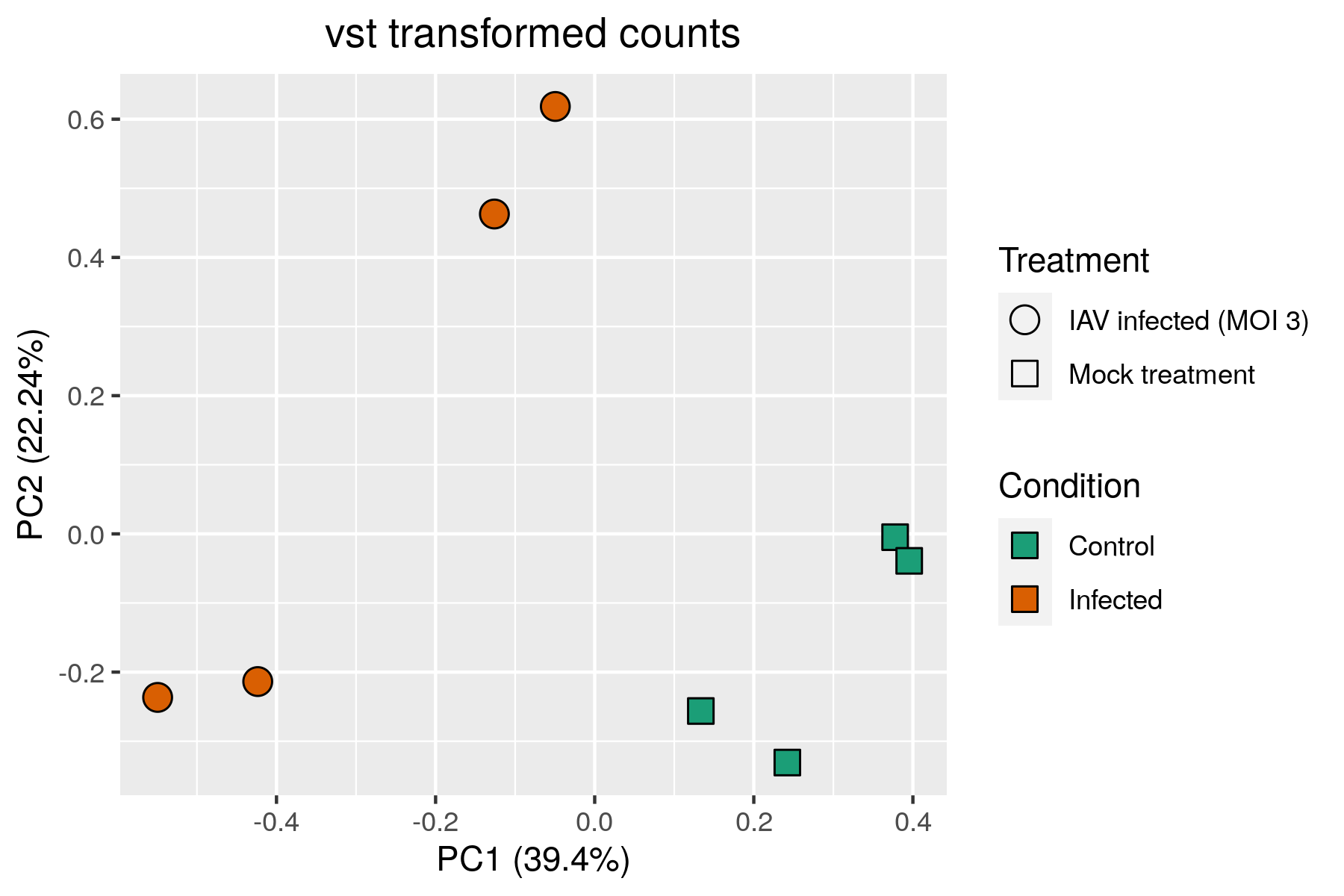

Supplement: Supplementary file 5 — Supplementary Data 3 [file 42003_2021_2095_MOESM5_ESM.zip › GSE147507_MainDataset/Series9_IAV/PCA_ConditionTreatment.png]

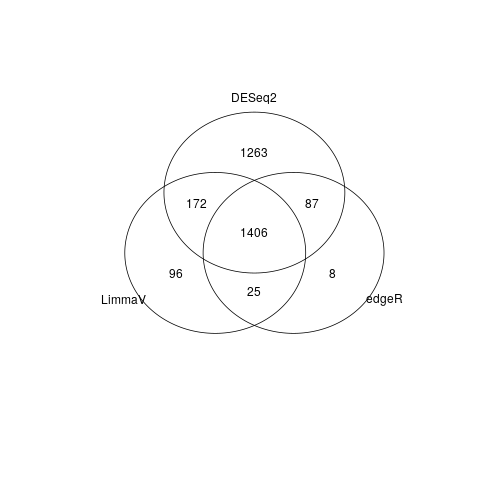

Supplement: Supplementary file 5 — Supplementary Data 3 [file 42003_2021_2095_MOESM5_ESM.zip › GSE147507_MainDataset/Series9_IAV/des_Condition/ControlVsInfected_edgeR-DESeq2-limmaVoom_venn.png]

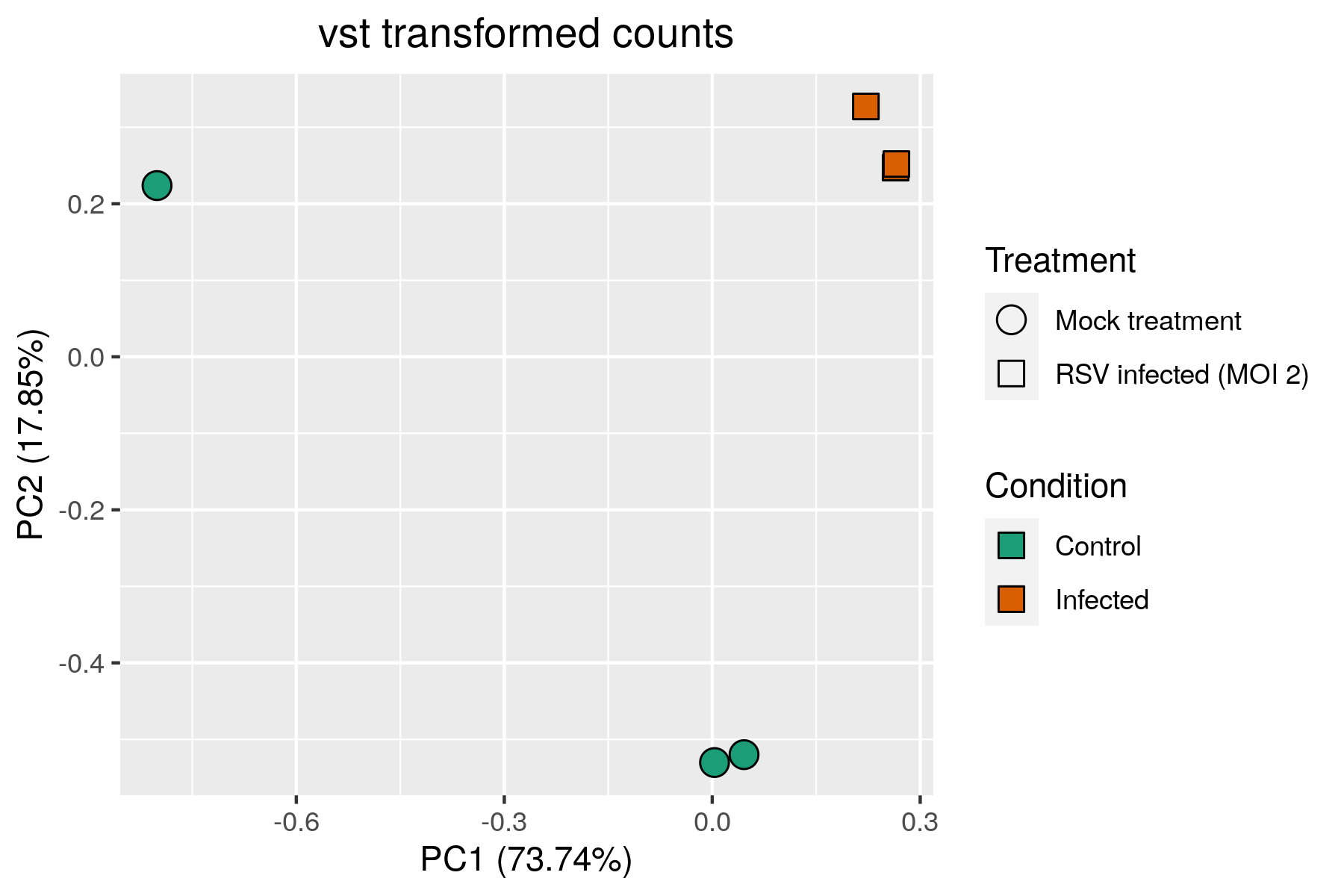

Supplement: Supplementary file 5 — Supplementary Data 3 [file 42003_2021_2095_MOESM5_ESM.zip › GSE147507_MainDataset/Series8_RSV/PCA_ConditionTreatment.png]

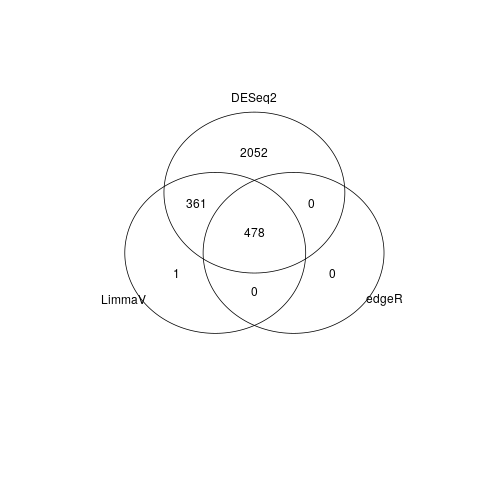

Supplement: Supplementary file 5 — Supplementary Data 3 [file 42003_2021_2095_MOESM5_ESM.zip › GSE147507_MainDataset/Series8_RSV/des_Condition/ControlVsInfected_edgeR-DESeq2-limmaVoom_venn.png]

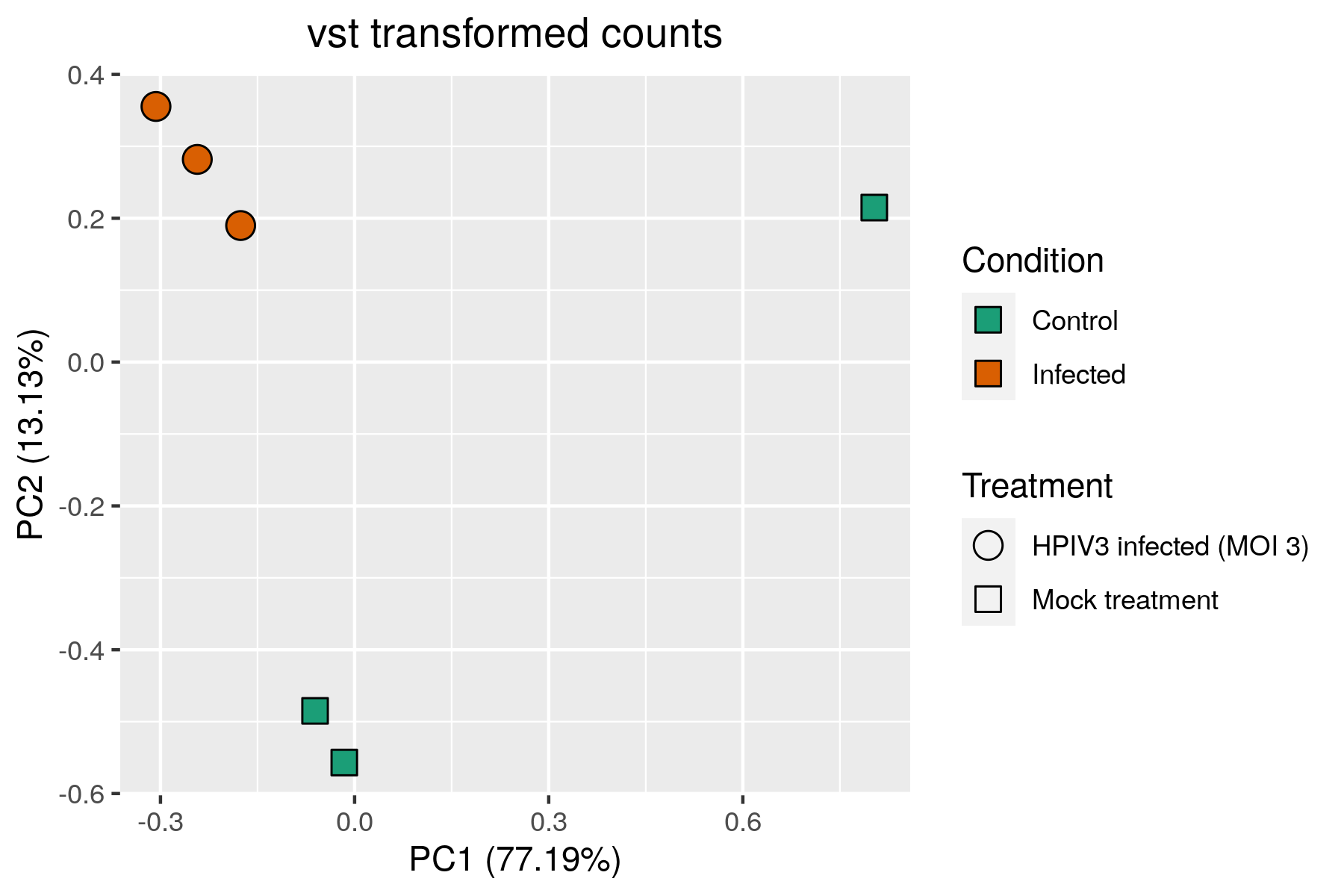

Supplement: Supplementary file 5 — Supplementary Data 3 [file 42003_2021_2095_MOESM5_ESM.zip › GSE147507_MainDataset/Series8_HPIV3/PCA_ConditionTreatment.png]

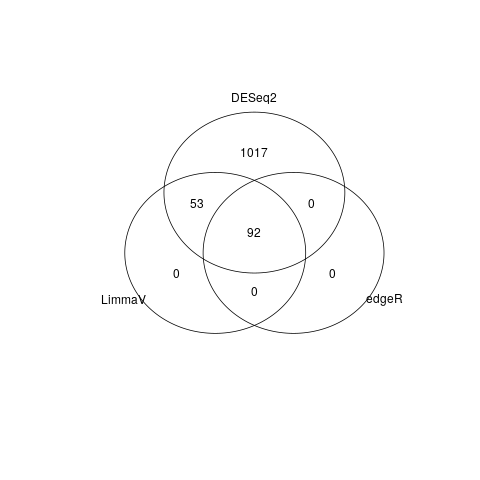

Supplement: Supplementary file 5 — Supplementary Data 3 [file 42003_2021_2095_MOESM5_ESM.zip › GSE147507_MainDataset/Series8_HPIV3/des_Condition/ControlVsInfected_edgeR-DESeq2-limmaVoom_venn.png]

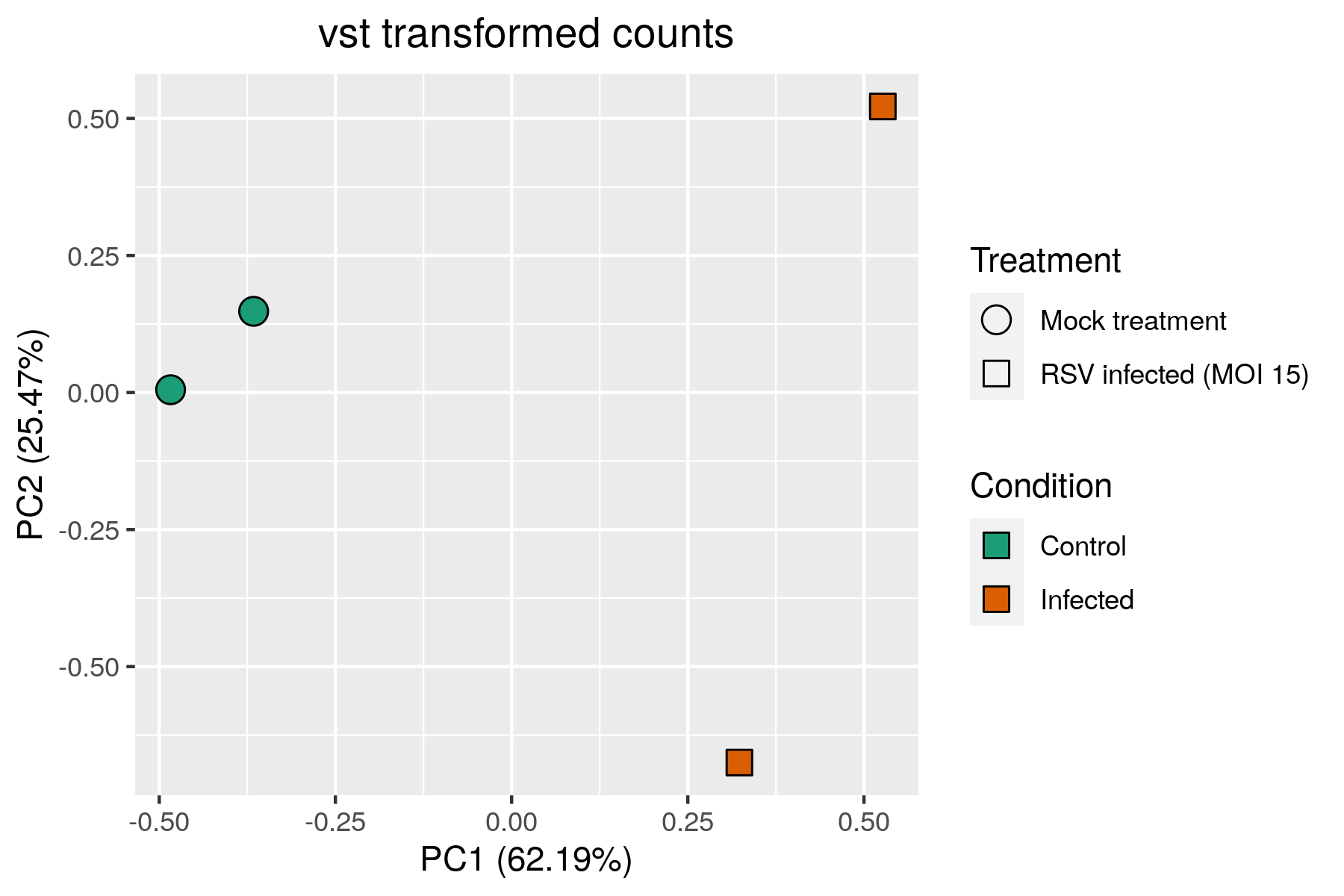

Supplement: Supplementary file 5 — Supplementary Data 3 [file 42003_2021_2095_MOESM5_ESM.zip › GSE147507_MainDataset/Series3_RSV/PCA_ConditionTreatment.png]

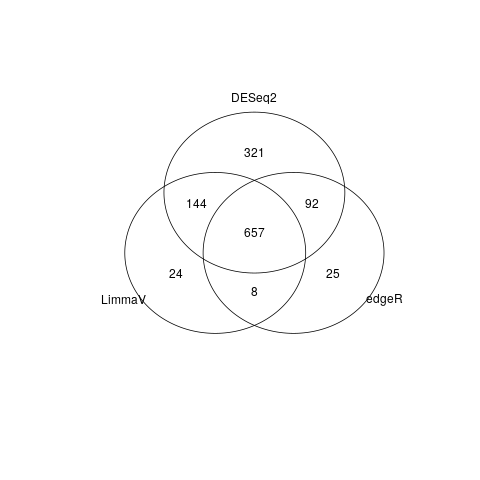

Supplement: Supplementary file 5 — Supplementary Data 3 [file 42003_2021_2095_MOESM5_ESM.zip › GSE147507_MainDataset/Series3_RSV/des_Condition/ControlVsInfected_edgeR-DESeq2-limmaVoom_venn.png]

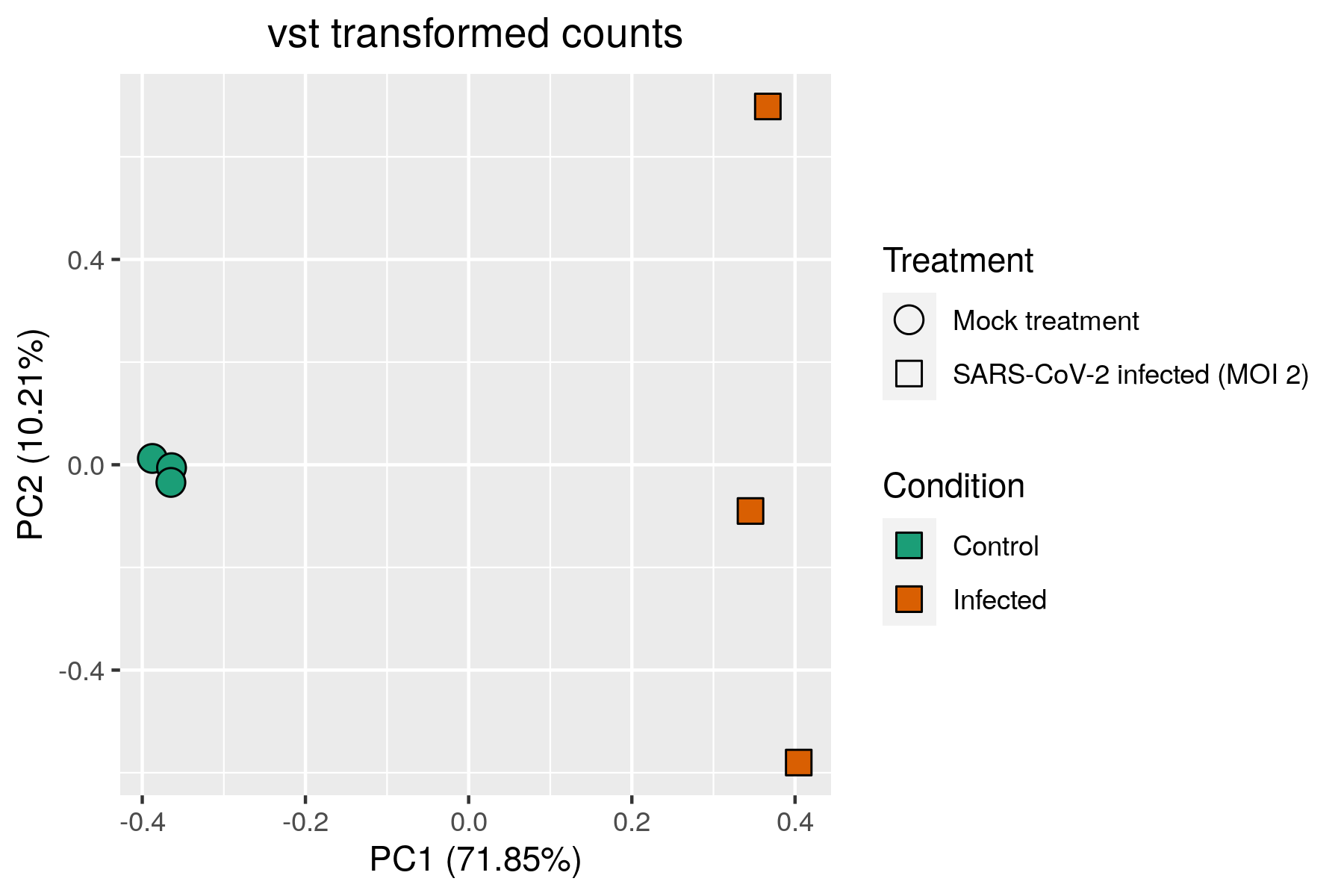

Supplement: Supplementary file 5 — Supplementary Data 3 [file 42003_2021_2095_MOESM5_ESM.zip › GSE147507_MainDataset/Series5_SARS-CoV-2/PCA_ConditionTreatment.png]

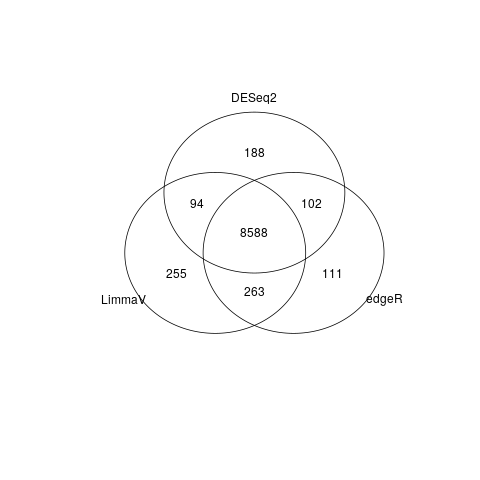

Supplement: Supplementary file 5 — Supplementary Data 3 [file 42003_2021_2095_MOESM5_ESM.zip › GSE147507_MainDataset/Series5_SARS-CoV-2/des_Condition/ControlVsInfected_edgeR-DESeq2-limmaVoom_venn.png]
