# Supplementary material for: Genome-wide bioinformatic analyses predict key host and viral factors in SARS-CoV-2 pathogenesis
Source: Commun Biol. 2021 May 17;4:590. doi: 10.1038/s42003-021-02095-0 (PMC8128904; doi:10.1038/s42003-021-02095-0)
Supplement: Supplementary file 21 — Reporting Summary [file 42003_2021_2095_MOESM21_ESM.pdf]

nature research

Corresponding author(s): Vanessa Aguiar-Pulido
Brett E. Pickett

Last updated by author(s): Mar 12, 2021

Reporting Summary

Nature Research wishes to improve the reproducibility of the work that we publish. This form provides structure for consistency and transparency in reporting. For further information on Nature Research policies, see our Editorial Policies and the Editorial Policy Checklist.

Statistics

For all statistical analyses, confirm that the following items are present in the figure legend, table legend, main text, or Methods section.

n/a	Confirmed	
☐	☒	The exact sample size (*n*) for each experimental group/condition, given as a discrete number and unit of measurement
☐	☒	A statement on whether measurements were taken from distinct samples or whether the same sample was measured repeatedly
☐	☒	The statistical test(s) used AND whether they are one- or two-sided<br>*Only common tests should be described solely by name; describe more complex techniques in the Methods section.*
☐	☒	A description of all covariates tested
☐	☒	A description of any assumptions or corrections, such as tests of normality and adjustment for multiple comparisons
☒	☐	A full description of the statistical parameters including central tendency (e.g. means) or other basic estimates (e.g. regression coefficient) AND variation (e.g. standard deviation) or associated estimates of uncertainty (e.g. confidence intervals)
☒	☐	For null hypothesis testing, the test statistic (e.g. *F*, *t*, *r*) with confidence intervals, effect sizes, degrees of freedom and *P* value noted<br>*Give P values as exact values whenever suitable.*
☒	☐	For Bayesian analysis, information on the choice of priors and Markov chain Monte Carlo settings
☒	☐	For hierarchical and complex designs, identification of the appropriate level for tests and full reporting of outcomes
☒	☐	Estimates of effect sizes (e.g. Cohen's *d*, Pearson's *r*), indicating how they were calculated

Our web collection on statistics for biologists contains articles on many of the points above.

Software and code

Policy information about availability of computer code

Data collection	RNA-seq data (GSE147507 and GSE150316) were downloaded from the Gene Expression Omnibus (GEO) database, hosted at the National Center for Biotechnology Information (NCBI). Normalized gene expression values in human lung tissue were obtained from the GTEx database, version 8 (https://gtexportal.org/home/datasets). Single-cell RNA-seq data for human lung cells were obtained from the NCBI GEO database under accession GSE122960. The reference genome sequences of SARS-CoV-2 (NC_045512), RaTG13 (MN996532.1), and SARS-CoV (NC_004718.3) were also downloaded from NCBI. Additionally, a list of known RNA-binding proteins (RBPs) and their Position Weight Matrices (PWMs) were downloaded from ATtRACT (https://attract.cnic.es/download). Finally, all SARS-CoV-2 complete genomes collected from humans that had disease severity information were downloaded from GISAID (https://www.gisaid.org/) on 11 November, 2020.
Data analysis	For the first RNA-seq dataset, data was downloaded from SRA using sra-tools (v2.10.8) and transformed to FASTQ with fastq-dump. FastQC (v0.11.9) and MultiQC (v1.9) were employed to assess the quality of the data used and the need to trim reads and/or remove adapters. Selected datasets were mapped to the human reference genome (GENCODE Release 19, GRCh37.p13) utilizing STAR (v2.7.3a). SAM files were converted to BAM files employing samtools (v1.9). Read quantification was performed using StringTie (v2.1.1) and the output data was postprocessed with an auxiliary Python script provided by the same developers to produce files ready for subsequent downstream analyses. DESeq2 (v1.26.0) was used for both datasets to identify differentially expressed genes (DEGs). The GOstats package (v2.54.0) and REVIGO were used to perform Gene Ontology enrichment analyses. Pathway enrichment analyses were carried out using the SPIA algorithm and DAVID (v6.8). To perform the integration of transcriptomic analysis with the human metabolic network, Recon (v2.04), EBSeq (v1.28) and the Moomin method were used. The isoform analysis was carried out utilizing the IsoformSwitchAnalyzeR R package (v1.11.3), the Coding-Potential Assessment Tool (CPAT) , IUPred2 , SignalP and Pfam tools. The TEtools software was employed to perform the transposable element (TE) analysis. Custom code was developed to carry out the RBP analysis, available at https://github.com/vaguiarpulido/covid19-research/tree/master/scripts/rbp. Finally, the viral genotype-phenotype correlation analysis was performed utilizing MAFFT (v7.464) and the meta-CATS algorithm (https://github.com/bpickett/megaCATS).

For manuscripts utilizing custom algorithms or software that are central to the research but not yet described in published literature, software must be made available to editors and reviewers. We strongly encourage code deposition in a community repository (e.g. GitHub). See the Nature Research guidelines for submitting code & software for further information.

Data

Policy information about availability of data

All manuscripts must include a data availability statement. This statement should provide the following information, where applicable:

- Accession codes, unique identifiers, or web links for publicly available datasets
- A list of figures that have associated raw data
- A description of any restrictions on data availability

All data used in this analysis were downloaded from publicly available servers.

Field-specific reporting

Please select the one below that is the best fit for your research. If you are not sure, read the appropriate sections before making your selection.

☒ Life sciences ☐ Behavioural & social sciences ☐ Ecological, evolutionary & environmental sciences

For a reference copy of the document with all sections, see nature.com/documents/nr-reporting-summary-flat.pdf

Life sciences study design

All studies must disclose on these points even when the disclosure is negative.

Sample size	We employed data from the only published study available to date in which cells or patient tissue were infected with SARS-CoV-2 along with other viruses. Other studies have focused on the sequencing of fluids (blood, bronchoalveolar lavage fluid), and may not reflect directly the cellular response to this virus.
Data exclusions	Sequencing quality was assessed and lung biopsies were excluded from GSE147507 due to low alignment percentage to the human reference genome. Principal component analysis was performed on the transformed values obtained after applying the variance stabilizing transformation to remove outliers from GSE150316.<br>For the viral genotype-phenotype correlation analysis, SARS-CoV-2 genomes from GISAID responsible for introducing excessive gaps in the initial alignment employing MAFFT were identified and removed.
Replication	Publications verifying some of the findings presented in this study were found and cited.
Randomization	For the viral genotype-phenotype correlation analysis, as a further validation step, we performed the same analysis comparing viral sequence variants against potential confounders, such as the biological sex or age group of the patients.
Blinding	N/A

Reporting for specific materials, systems and methods

We require information from authors about some types of materials, experimental systems and methods used in many studies. Here, indicate whether each material, system or method listed is relevant to your study. If you are not sure if a list item applies to your research, read the appropriate section before selecting a response.

Materials & experimental systems

n/a	Involved in the study	
☒	☐	Antibodies
☒	☐	Eukaryotic cell lines
☒	☐	Palaeontology and archaeology
☒	☐	Animals and other organisms
☒	☐	Human research participants
☒	☐	Clinical data
☒	☐	Dual use research of concern

Methods

n/a	Involved in the study	
☒	☐	ChIP-seq
☒	☐	Flow cytometry
☒	☐	MRI-based neuroimaging
